# Comparison of Five Near-Infrared Fluorescent Folate Conjugates in an Ovarian Cancer Model

**DOI:** 10.1007/s11307-021-01685-y

**Published:** 2021-12-10

**Authors:** Elvira García de Jalón, Katrin Kleinmanns, Vibeke Fosse, Ben Davidson, Line Bjørge, Bengt Erik Haug, Emmet McCormack

**Affiliations:** 1 Centre for Cancer Biomarkers CCBIO, Department of Clinical Science, The University of Bergen, Jonas Lies vei 65, 5021 Bergen, Norway; 2 Department of Chemistry and Centre for Pharmacy, University of Bergen, Allégaten 41, N-5007 Bergen, Norway; 3 Department of Pathology, Oslo University Hospital, Norwegian Radium Hospital, and Faculty of Medicine, Institute of Clinical Medicine, University of Oslo, Oslo, Norway; 4 Department of Obstetrics and Gynaecology, Haukeland University Hospital, 5021 Bergen, Norway; 5 Centre for Pharmacy, Department of Clinical Science, The University of Bergen, Jonas Lies vei 65, 5021 Bergen, Norway; 6 Vivarium, Department of Clinical Science, The University of Bergen, Jonas Lies vei 65, 5021 Bergen, Norway

**Keywords:** Folate, Near-infrared fluorescence imaging, NIRF, Intraoperative navigation, Image-guided surgery, Ovarian, NIR dyes, Pharmacokinetics, Biodistribution, Folate receptor alpha

## Abstract

**Purpose:**

Fluorescence imaging (FLI) using targeted near-infrared (NIR) conjugates aids the detection of tumour lesions pre- and intraoperatively. The optimisation of tumour visualisation and contrast is essential and can be achieved through high tumour-specificity and low background signal. However, the choice of fluorophore is recognised to alter biodistribution and clearance of conjugates and is therefore a determining factor in the specificity of target binding. Although ZW800-1, IRDye® 800CW and ICG are the most commonly employed NIR fluorophores in clinical settings, the fluorophore with optimal *in vivo* characteristics has yet to be determined. Therefore, we aimed to characterise the impact the choice of fluorophore has on the biodistribution, specificity and contrast, by comparing five different NIR fluorophores conjugated to folate, in an ovarian cancer model.

**Procedures:**

ZW800-1, ZW800-1 Forte, IRDye® 800CW, ICG-OSu and an in-house synthesised Cy7 derivative were conjugated to folate through an ethylenediamine linker resulting in conjugates **1**–**5**, respectively. The optical properties of all conjugates were determined by spectroscopy, the specificity was assessed *in vitro* by flow cytometry and FLI, and the biodistribution was studied *in vivo* and *ex vivo* in a subcutaneous Skov-3 ovarian cancer model.

**Results:**

We demonstrated time- and receptor-dependent binding of folate conjugates *in vitro* and *in vivo*. Healthy tissue clearance characteristics and tumour-specific signal varied between conjugates **1**–**5**. ZW800-1 Forte (**2**) revealed the highest contrast in folate receptor alpha (FRα)-positive xenografts and showed statistically significant target specificity. While conjugates **1**, **2** and **3** are renally cleared, hepatobiliary excretion and no or very low accumulation in tumours was observed for **4** and **5**.

**Conclusions:**

The choice of fluorophore has a significant impact on the biodistribution and tumour contrast. ZW800-1 Forte (**2**) exhibited the best properties of those tested, with significant specific fluorescence signal.

**Supplementary Information:**

The online version contains supplementary material available at 10.1007/s11307-021-01685-y.

## Introduction

Fluorescence imaging (FLI) has developed into a key tool for monitoring tumour progression, invasion and treatment efficacy in preclinical xenograft models [1, 2]. In addition, FLI facilitates biological assessment and thus aids the clinical translation of newly developed fluorescent dyes and their corresponding conjugates [3, 4] for intra-operative tumour visualisation, often referred to as fluorescence image-guided surgery (FIGS) [5]. Complete tumour resection remains a cornerstone in cancer therapy and is strongly associated with decreased local recurrence rates and prolonged survival [6, 7]. Using target-specific fluorescent conjugates, which are composed of a targeting ligand, a linker and a fluorophore, FIGS aims to illuminate diseased tissue, small metastases and vital structures in real time. Thus, FIGS offers enormous potential to assist the surgeon with tumour visualisation, and identification of additional metastases and margins to ultimately achieve complete resection [8, 9]. A critical aspect for the success of this technique is a high tumour-to-background ratio (TBR), which provides a good contrast between the tumour mass and healthy tissues.

Much effort has been dedicated to identifying tumour-specific targets and fluorescent conjugates that could be explored clinically. Folate receptor alpha (FRα) is an example of a widely studied tumour-associated molecular target, which is overexpressed in up to 90% of epithelial ovarian cancers (EOC) [10, 11], among others. Thus, FRα-targeting by folate is a good model system for evaluating FLI in EOC. The application of FRα-targeting in preclinical molecular imaging demonstrated promising results [12], and has since been clinically assessed for molecular therapy [13, 14], in addition to its application in late stage clinical trials for FIGS of several cancers [8, 15]. Coupled with the identification of novel targets, the development of promising targeting ligands has flourished in recent years, including small molecules, peptides, antibodies or antibody fragments [16, 17]. The ideal fluorescent conjugate needs to exhibit low toxicity, high photochemical stability and fast clearance. To accomplish this, the design of fluorescent conjugates requires special attention, not only in the choice of targeting ligand, but also in the selection of fluorophore and linker. However, as discussed by Debie *et al.* [16], most of these targeting ligands are often conjugated to readily available fluorophores, such as IRDye® 800CW. Fluorophores with emission in the near-infrared region (NIR; 650–900 nm) are preferred due to reduced tissue absorption and increased depth penetration of light. The NIR dye indocyanine green (ICG) has been employed for the assessment of vital structures such as bile ducts location during surgery and for the visualisation of ovarian tumour tissue by passive accumulation [18, 19] and/or by enhanced permeability and retention (EPR) effect [20, 21]. Due to its clinical approval, ICG has also been employed in targeted approaches [22, 23], although its use has not been extensively accepted due to the challenges with conjugation of this fluorescent dye to targeting ligands. Moreover, the fluorescence of ICG is known to quench after conjugation to proteins, which may have a detrimental impact on its use for imaging purposes [24]. Besides IRDye® 800CW and ICG, other commercially available NIR dyes are in different stages of development for FIGS [16, 17]. Various fluorophores are based on the cyanine scaffold, and are decorated with a variety of functional groups, which results in different pharmacological properties [25]. Such differences in physicochemical properties of the NIR fluorophores also have a major impact on the target binding specificity of their conjugates since, e.g. biodistribution and clearance can be affected with several cases previously reported where the choice of fluorophore, or linker, affected the affinity of the targeting ligand to the molecular target of interest [26–28]. Those studies highlight the importance of reducing background signal, improving contrast during FLI by avoiding non-specific binding, improving stability and enhancing clearance from the body.

**Fig. 1.**
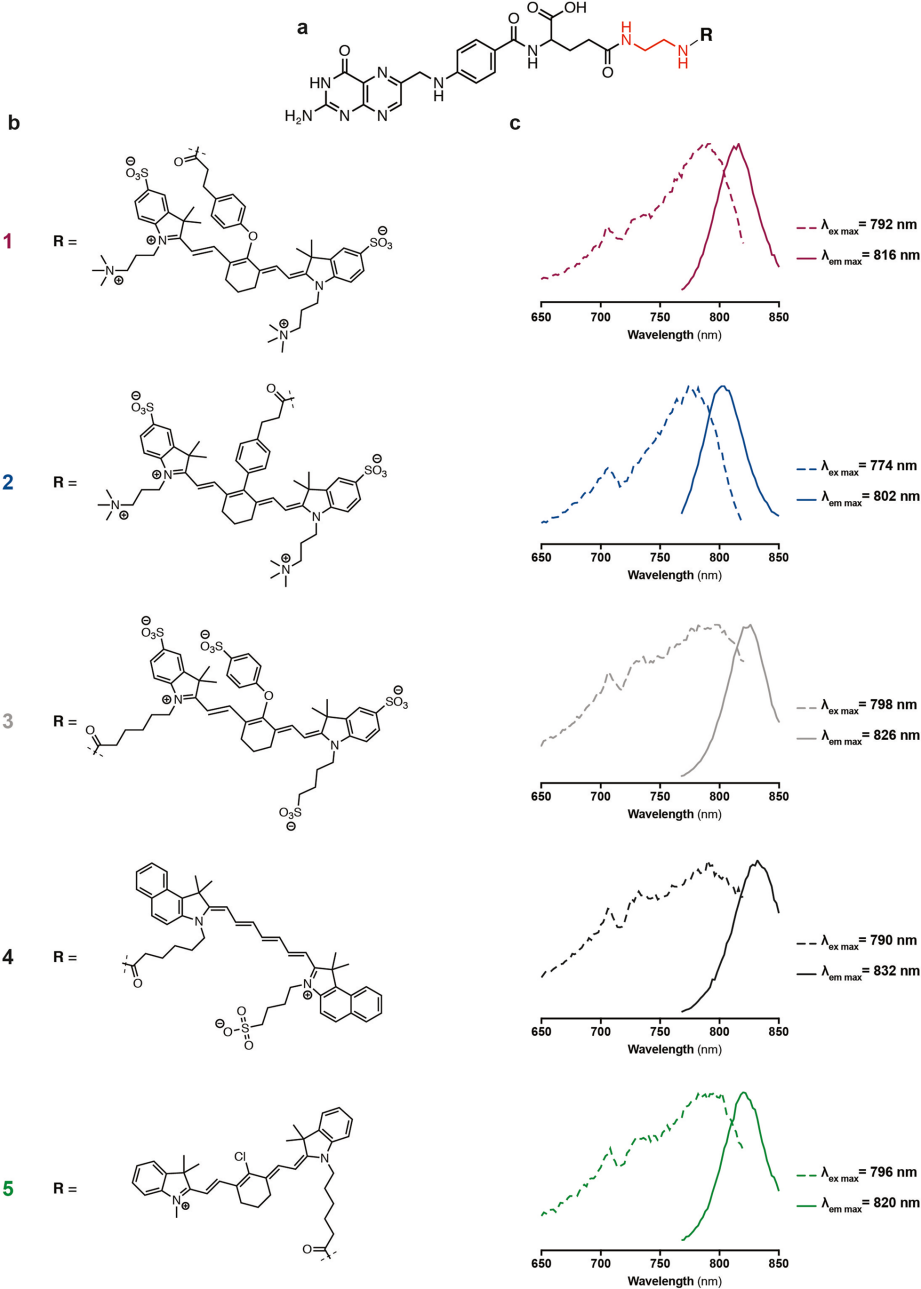
Chemical structure and optical properties of five far-red shifted folate conjugates. All conjugates were synthesised to target the FRα through the folate ligand. (a) The conjugation of folate (depicted in black) through an ethylenediamine linker (EDA, depicted in red) to (b) five fluorescent dyes was performed. (c) Spectral properties including peak emission and excitation of conjugates **1**–**5** dissolved in DMSO.

Using folate as a ligand to target FR, we aimed to investigate the impact of NIR fluorophores on biodistribution, specificity and sensitivity in a comparison study of five fluorescent conjugates. We conjugated four commercially available fluorescent dyes with similar spectral properties and clinical translational potential, namely ZW800-1 (**1**), ZW800-1 Forte (**2**), IRDye® 800CW (**3**) and ICG-OSu (**4**), and one in-house synthesised Cy7 dye (**5**), to folate through an ethylenediamine (EDA) linker (see Fig. 1 for structures) and evaluated the different conjugates in subcutaneous EOC xenograft models.

## Materials and Methods

### Synthetic Procedures

γ-Ethylenediamine folate (EDAF) was reacted with the NHS esters of ZW800-1 (Curadel LCC, MA, USA), ZW800-1 Forte (Curadel LCC), IRDye® 800CW (LI-COR, NE, USA), ICG-OSu (AAT Bioquest, CA, USA) and an in-house synthesised Cy7 derivative, to give conjugates **1**–**5**, respectively. Detailed synthetic and analytical data are given in the electronic supplementary material.

### Fluorescence Spectroscopy

The spectral characteristics of the conjugates **1**–**5** were determined using a Spark 20M plate reader (Tecan, Switzerland). The emission and excitation of all conjugates were measured in triplicates with a bandwidth of 5 nm in a 96-well Half-Area Microplate at a concentration of 100 μM dissolved in phosphate buffered saline (PBS). The fluorescence intensity was plotted as normalised relative fluorescence units (RFU).

### Cell Lines and Cell Culture

Human EOC Skov-3, lung carcinoma A549 and cervical adenocarcinoma HeLa cell lines were employed to study FRα expression. Cells were cultured in DMEM (Sigma-Aldrich, MO, USA) supplemented with 10% FBS and 1% L-glutamine (Sigma-Aldrich) and kept at 37 °C and 5% CO_2_. For the binding and specificity assay, cells were cultured in folic acid free medium.

### In Vitro Validation: Flow Cytometry, Optical FLI and Cytotoxicity

The degree of FRα binding in Skov-3, A549 and HeLa cells was quantitatively determined and expressed as antibody-binding capacity (ABC) units, employing a bead calibration kit (Qifikit, Dako Agilent, CA, USA). 1 × 10^5^ cells were re-suspended in 100 μL PBS + 2% bovine serum albumin (BSA) buffer and stained with a saturated concentration of 5 μg unconjugated mouse anti-human FRα (Clone 548908, Thermo Fisher Scientific, MA, USA) for 45 min at 4 °C. An unconjugated monoclonal CD33 IgG1 antibody (Clone WM53, 1:20 Bio-Rad, UK) was used as a negative control. Cells, set-up beads and calibration beads were each washed twice with PBS-BSA-Azide buffer and stained with a secondary FITC-conjugated antibody (provided in the Qifikit, 1:50) for 45 min at 4 °C. After two further washing steps, samples were acquired and recorded with the BD Fortessa flow cytometer (BD Biosciences, NJ, USA), with a laser excitation at 488 nm and BP emission filter at 530 ± 15 nm.

The binding and specificity to FRα of conjugates **1**–**5** was determined by FLI in (1) FRα low, intermediate and high expressing cell lines and (2) different conjugate concentrations with or without the addition of folic acid (FA). First, 5 × 10^6^ A549 (negative control), HeLa (positive control) and Skov-3 cells were incubated with 10 μM of conjugate **1**–**5.** Secondly, 7.5 × 10^5^ HeLa cells were incubated with 10, 1 or 0.1 μM of conjugates **1**–**5** in the presence or absence of 100-fold molar excess of FA. For both experiments, cells were incubated for 4 h at 37 °C, washed twice, centrifuged and kept in cell pellets without supernatant. Fluorescence images were acquired with the IVIS Spectrum In Vivo Imaging System (PerkinElmer Inc., MA, USA) using the filter settings λ_ex_ = 745 ± 15 nm, λ_em_ = 800 ± 10 nm and signals were expressed as average radiance (p/s/cm^2^/sr).

The toxicity of all five conjugates was tested *in vitro*. Skov-3 cells were cultured in triplicate and incubated for 4 h with 0.1–100 μM of each conjugate, before cell proliferation was assessed using a water-soluble tetrazolium salt assay (WST-1, Roche applied Science, Germany). The metabolite formazan was measured spectrophotometrically to determine the metabolically active cells in each well. The formazan concentration of each sample was normalised to Skov-3 cells cultured without the addition of a conjugate.

### Subcutaneous Xenograft Models

All applicable institutional and/or national guidelines for the care and use of animals were followed. All experiments were approved by The Norwegian Animal Research Authority and conducted according to The European Convention for the Protection of Vertebrates Used for Scientific Purposes (Application ID 14128). NOD-*scid* IL2Rg^null^ mice (referred to as NSG) were housed in groups of ≤ 5 in individually ventilated cages changed twice per month and kept in a 12-h dark/night schedule at 21 °C at the Vivarium, University of Bergen. Observations for general condition and body weights were recorded twice per week and, when indicated, the animals were euthanised according to institutional guidelines. Skov-3 and A549 subcutaneous xenografts were established through bi-lateral flank injections of 5 × 10^6^ cells, resuspended in 100 μL PBS containing 16.7% Matrigel (Corning Inc., MA, USA). Tumour volumes were measured weekly with a digital calliper and calculated using the ellipsoid volume formula: Volume = π (length × width × height)/6.

### Optical Imaging of the Subcutaneous Xenografts

#### *In Vivo* Biodistribution and Optimal Time Point Imaging

For biodistribution assessment of conjugates **1**–**5**, mice were intravenously (iv) injected with 100 μL of a 10-μM conjugate solution when mean tumour volumes reached ≈150 mm^3^, corresponding to a dose of 500 nmol/mouse. The dose was chosen on the basis of preclinical mouse models previously performed using folate-NIR (OTL38) FLI [28]. To determine the timepoint for the optimal TBR, optical imaging was performed at 0 h, 0.5 h, 1 h, 2 h, 4 h, 6 h, 8 h, 24 h and 48 h using the IVIS Spectrum. Fluorescence signals were analysed with the Living Imaging® software v4.5 (PerkinElmer Inc.) and expressed as average radiance (p/s/cm^2^/sr). Regions of interest (ROI) were manually drawn around the subcutaneous tumours in the flanks and the adjacent tissue to calculate TBRs at all time points. Next, subcutaneous xenograft models were imaged at the pre-determined optimal time points, which were 4 h for conjugates **1**, **2** and **3** and 24 h for conjugates **4** and **5**.

#### *Ex Vivo* Biodistribution

Organ biodistribution was assessed *ex vivo* for each conjugate in Skov-3 and in the control cell line, A549. Four hours after iv administration of **1**, **2** and **3** and 24 h after injection of **4** and **5** (100 μl, 10 μM), mice were euthanised and tumours and organs were harvested. FLI was performed with the IVIS Spectrum, and subsequently, fluorescence was acquired using the FLARE® intraoperative system (Curadel LLC), employing the NIR channel #2 (λ_ex_ = 760 ± 3 nm and λ_em_ = > 781 nm longpass) at three different exposure times (500, 1000 and 2000 ms) at a gain of 1. IVIS Spectrum data were analysed as previously described, and FLARE data were analysed using ImageJ (Fiji, version 1.52p, [29]) and the integrated FLARE analysis platform using grey scale values expressed in arbitrary units (AU).

### Immunohistochemistry

Resected tumours of A549 and Skov-3 were fixed in 4% paraformaldehyde, embedded in paraffin(FFPE) and immunohistochemically stained as described earlier [30]. In short, 5-μm sectioned FFPE samples were stained with anti-human FRα (clone SA170417DD, 1:2000, Invitrogen) and evaluated using an immunoreactivity scoring system defined by the product (I*E) with I being the staining intensity (0 = negative, 1 = weak, 2 = moderate) and E being the staining extent (0 = 0%, 1 = 1–5%, 2 = 6–25%, 3 = 26–75%, 4 = 76–100%) resulting in a score of 0–8. Rabbit-specific polymer-horseraddish peroxidase was used to avoid false-positive cross-specific bindings in mouse tissue.

### Statistics

Results are given as mean ± standard deviation (SD). After randomisation, a one-way ANOVA was applied to ensure unbiased assignment of tumour volumes among the experimental groups. Comparison of means was performed using Student *t*-tests. All statistical tests were performed using GraphPad Prism v 6.0h (GraphPad Software Inc, CA, USA) and *p* < 0.05 was considered significant.

## Results

### Synthesis, Purification and Optical Properties Determination of Cy7 Conjugates

Fluorophore choice is known to influence the tumour-specific fluorescence signal due to unspecific accumulation and distribution in the body [26]. To investigate which fluorescent dye favours tumour-specific optical imaging in EOC xenograft models, we conjugated five different fluorophores (the commercially available ZW800-1, ZW800-1 Forte, IRDye® 800CW and ICG-OSu, and one in-house synthesised Cy7) to the well-established targeting ligand folate, utilising an ethylenediamine (EDA) linker. The chemical structure of the resulting five different fluorescent conjugates, **1**–**5,** is depicted in Fig. 1b. Detailed descriptions of the different synthetic approaches performed to obtain the targeting ligand and conjugates **1**–**5**, as well as the corresponding analytical data (Fig. S1 to S15), logD and surface charge distribution (Fig. S16), can be found in the electronic supplementary material.

Using conjugates **1**–**5**, we evaluated how the conjugation of the different fluorophores to EDAF (Fig. 1a) affected the optical properties. To do so, we defined their individual excitation and emission spectra, and determined the optimal FLI parameters. The different conjugates (Fig. 1b) were diluted in medium or in DMSO and the peak excitation and emission obtained are reported in Table 1, Fig. 1c and Fig. S17.

**Table 1:** Summary of the spectroscopic properties of conjugates **1–5** measured in medium and DMSO

Conjugate	Culture medium			DMSO		
	λ_ex_ (nm)	λ_em_ (nm)	Stokes shift (nm)	λ_ex_ (nm)	λ_em_ (nm)	Stokes shift (nm)
**1**	777	802	25	792	816	24
**2**	760	788	28	774	802	28
**3**	783	808	25	798	826	28
**4**	784	820	36	790	832	42
**5**	789	804	15	796	820	24

### Folate Receptor Alpha Expression and Conjugate Binding

**Fig. 2.**
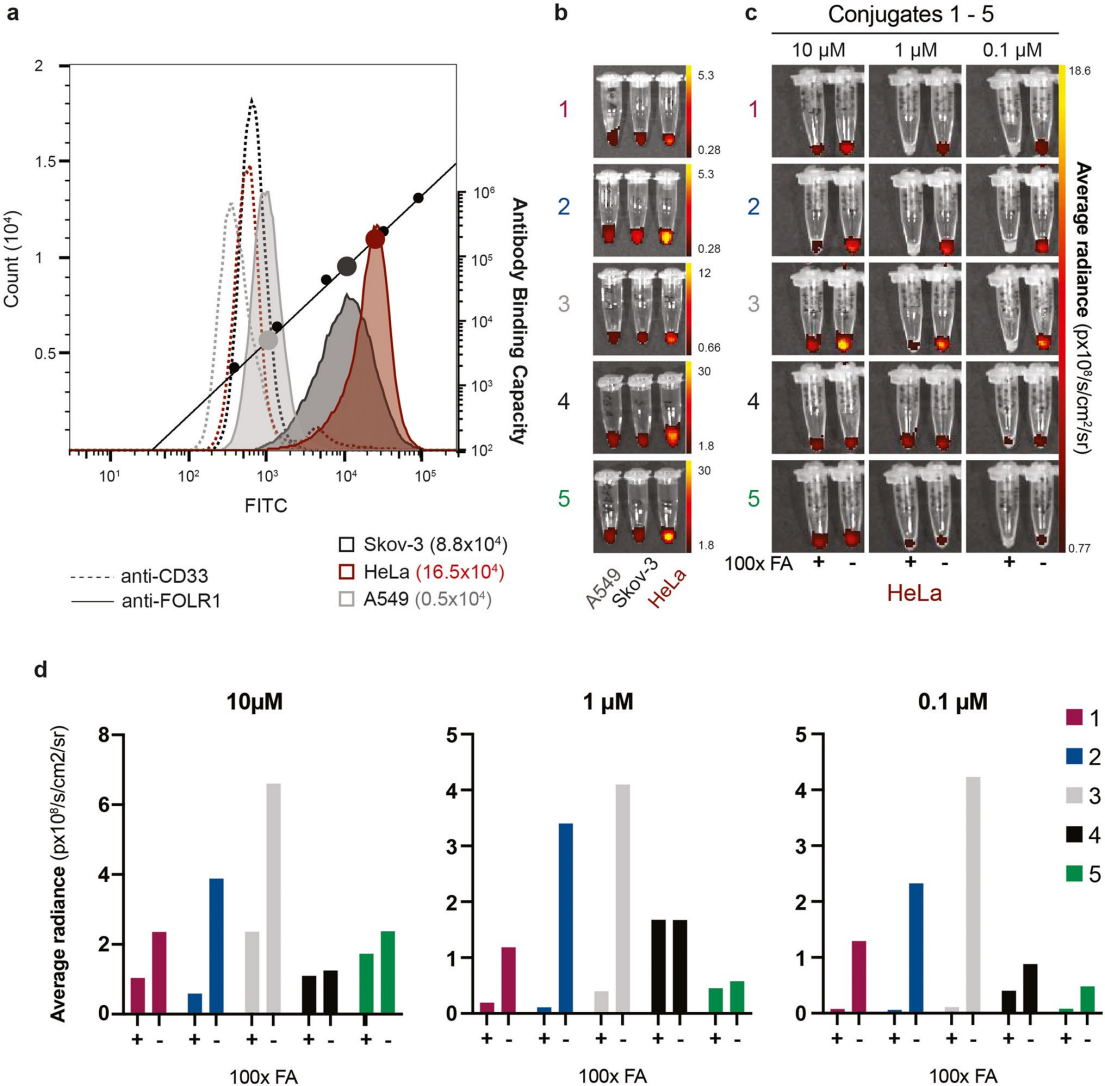
Folate receptor alpha (FRα) expression and conjugate binding affinity in three human tumour cell lines. **a** The antibody binding capacity of the ovarian cancer cell line Skov-3 was evaluated and compared to the FRα-positive control cell line HeLa and negative control cell line A549. Flow cytometry fluorescence intensity of anti-FOLR1-FITC cancer cell lines was correlated to the antibody binding capacity (ABC) of calibration beads. The resulting ABC was calculated and presented for each cell line. An anti-CD33 monoclonal antibody served as a negative control. **b** For the validation of target-specific binding of all five conjugates, stained cell line pellets (5 × 10^6^ cells) were imaged at the IVIS spectrum. Data reported as average radiance (p×10^8^/s/cm^2^/sr). **c** HeLa cell line pellets stained with 10, 1 and 0.1 μM of conjugates **1**–**5** in the presence or absence of 100-fold molar excess folic acid (FA) imaged at the IVIS spectrum. **d** Quantification (ROI) of fluorescence signals from (**c**) reported as average radiance (p×10^8^/s/cm^2^/sr).

Next, we investigated the expression of FRα in the EOC cell line Skov-3 prior to *in vivo* FLI. We quantified the FRα binding sites of Skov-3 using an antibody-based flow cytometry approach and compared it to the expression profiles of the highly FRα-positive endometrial cancer cell line HeLa and the FRα low expressing lung cancer cell line A549 (Fig. 2a). Skov-3 revealed an average expression of 8.8 × 10^4^ FRα ABC/cell, compared to the 0.5 × 10^4^ obtained for A549, confirming FRα abundance (Fig. 2a). For the evaluation of the specific binding capacity and possible steric interferences in the binding to the FRα after conjugation, we incubated 5 × 10^6^ cells per cell line with 10 μM of each conjugate (**1**–**5**) for 4 h. Fluorescence signals were obtained from all different cell line pellets at the IVIS Spectrum, with the highest fluorescence intensities exhibited by the HeLa cells for all five conjugates (Fig. 2b), consistent with the high FRα expression (16.5 × 10^4^ ABC/cell) (Fig. 2a). In addition, the specific binding of conjugates **1**–**5** was assayed in the presence and absence of 100-fold molar excess of folic acid (FA) at 10, 1 or 0.1 μM, using HeLa cells. In the presence of FA, the intensity of the fluorescence was greatly reduced, especially for the lowest concentrations (Fig. 2c, d and S18). In addition, no cytotoxicity or inhibition of cell proliferation was observed at the employed dose for any of the conjugates (Fig. S19).

### Biodistribution of Folate Conjugates in Skov-3 Subcutaneous Xenograft Model

**Fig. 3.**
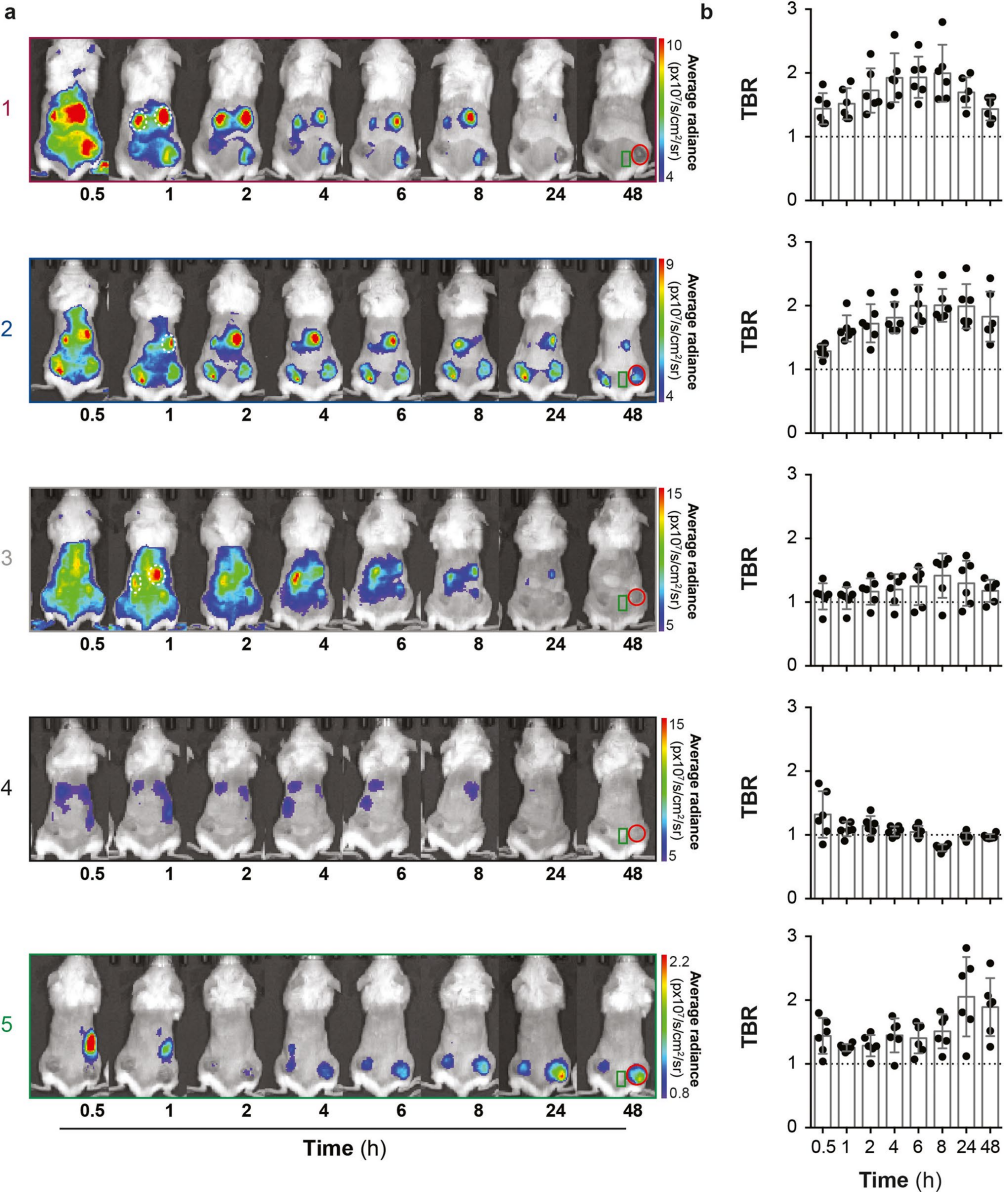
Assessment of conjugate biodistribution in subcutaneous Skov-3 xenograft models. (**a**) Longitudinal *in vivo* whole body optical FLI at eight different time points (0.5 h, 1 h, 2 h, 4 h, 6 h, 8 h, 24 h and 48 h) of one representative mouse (*n* = 3). The fluorescence signal in the tumours (red circle), surrounding tissue (green rectangle) and kidneys (dashed white circle, when required) of each conjugate **1**–**5** (which were based on ZW800-1, ZW800-1 Forte, IRDye® 800CW, ICG-OSu and one in-house synthesised dye, respectively) was quantified and (**b**) presented as tumour to background ratios (TBR) for all time points.

To assess the impact of the different fluorophores in conjugates **1**–**5** on the tumour-specificity and sensitivity, subcutaneously engrafted NSG mice (*n* = 3 per conjugate) were imaged at eight different time points from 0.5 to 48 h (Fig. 3a). Tumour-specific fluorescence was observed for all conjugates except **4** (Fig. 3a). Conjugate **2** exhibited an intense fluorescence signal in Skov-3 tumours early after iv injection that was sustained up to 48 h. High, non-specific accumulation in healthy tissue at early time points was observed for all conjugates, followed by different time- and excretion route-dependent elimination from the body. Mice injected with conjugates **1** and **3** exhibited residual fluorescence signal in the kidneys at 24 h (Fig. 3a white-dashed circles). For conjugates **4** and **5**, very low fluorescence intensity signals were obtained at any given time, with evidence of hepatic excretion (Fig. S20) observed as residual liver fluorescence for **4**. Interestingly, for conjugate **5,** the tumour fluorescence was not evident until 4 h post-injection, but then increased over time to reach the maximum TBR at 24 h post-injection. It should be noted that conjugate **1** employed for the *in vivo* experiments is estimated to have contained free dye in a ratio of 3:1 (conjugate **1**, free dye) (see Fig. S21). Following evaluation of conjugate biodistribution over time, the optimal time point for achieving the best TBR, expressed as the ratio between tumour-specific accumulation and surrounding fluorescence, was determined for each conjugate and was as follows: **1**, 8 h (TBR 2.0 ± 0.4); **2**, 8 h (TBR 2.0 ± 0.2); **3**, 8 h (TBR 1.4 ± 0.3); **4**, 0.5 h (TBR 1.3 ± 0.3); **5**, 24 h (TBR 2.1 ± 0.6) (Fig. 3b). Optimal time points at 8 h are unpractical for imaging purposes, limiting its applicability. For practicality, 4 h were selected for imaging, as acceptable TBR values were obtained at this time point, for conjugates **1** (TBR 1.9 ± 0.3), **2** (TBR 1.8 ± 0.2) and **3** (TBR 1.2 ± 0.2). For conjugates **4** (TBR 1.0 ± 0.1) and **5**, 24 h was chosen (Fig. 3b).

### Targeting Ligand Specificity and *Ex Vivo* Biodistribution

**Fig. 4.**
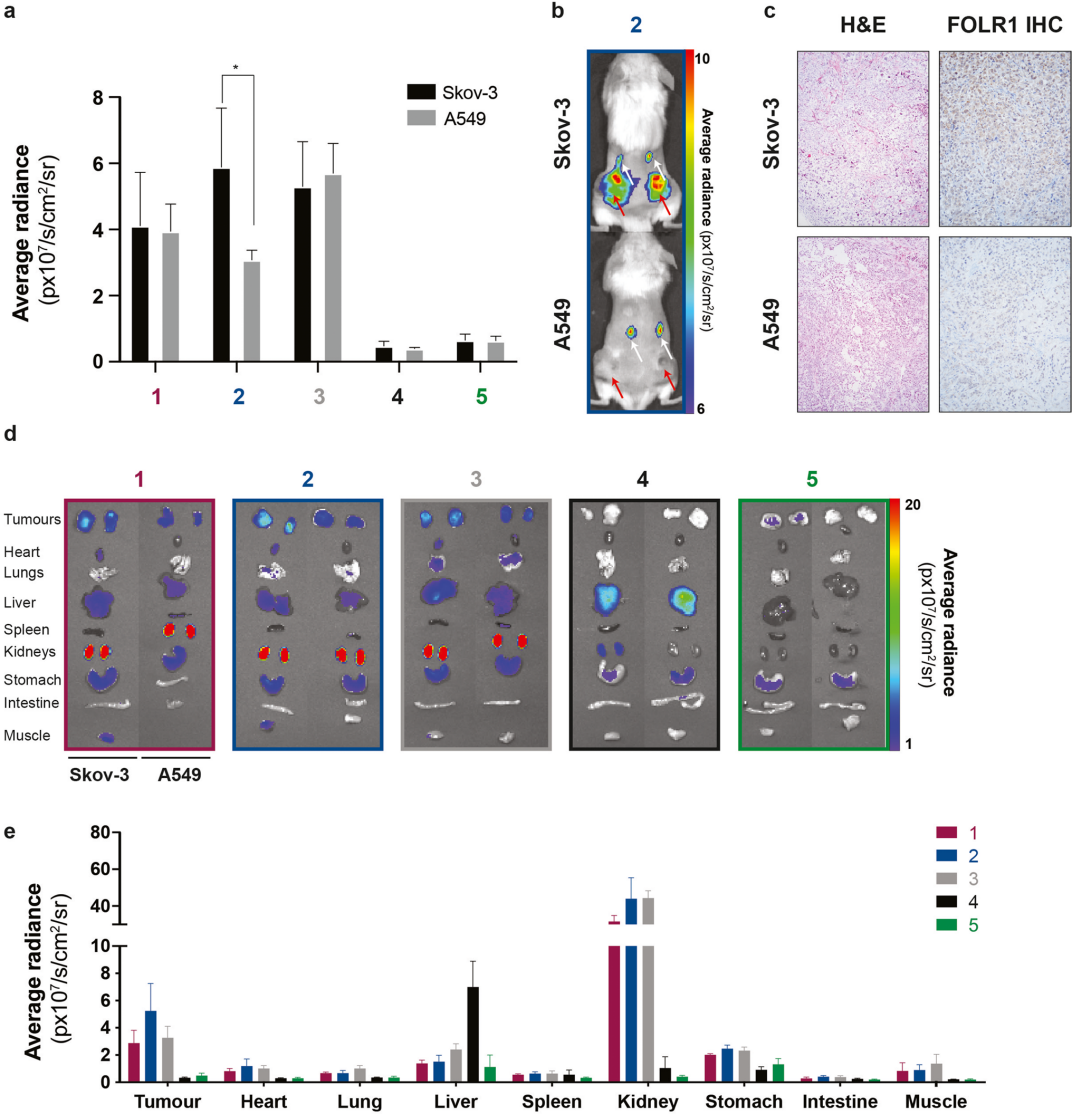
Quantification of average fluorescence intensities in Skov-3 and A549 subcutaneous xenografts. (**a**) By quantifying the *in vivo* fluorescence intensities of all five conjugates in Skov-3 (*n*= 8–14) and A549 (*n *= 2–4) at 4h for **1**–**3** and 24h for **4** and **5**, FRα expression-dependent accumulation in the tumour was observed. (**b**) Significant increased tumour fluorescence signal in Skov-3 compared to A549 4h after ZW800-1 Forte (**2**) injection. The fluorescence signal in tumours and in kidneys is highlighted with red and white arrows, respectively. (**c**) The expression and distribution of FRα were assessed by IHC in both xenografted cell line tumour samples (Skov-3 *n* = 12 and A549 *n* = 6). Sectioned tumour samples were additionally stained with haematoxylin and eosin. (**d**) *Ex vivo* tumour and organ average fluorescence intensities of Skov-3 and A549 were quantified (**e**) to assess the conjugate accumulation at the optimal time point determined for **1**–**5** (based on ZW800-1, ZW800-1 Forte, IRDye® 800CW, ICG-OSu and one in-house synthesised Cy7 dye, respectively). Statistical analysis (one-way ANOVA with unpaired Mann-Whitney *U* test) with *p* < 0,05 (*) was regarded as statistically significant.

To validate tumour-specific signal intensities, we assessed *in vivo* and *ex vivo* FLI in subcutaneous xenografts of Skov-3 and A549 at the optimal time points. Four hours after iv injection of conjugates **1**, **2** and **3**, and 24 h after administration of conjugates **4** and **5**, *in vivo* images were acquired for both groups using the IVIS Spectrum. As seen in Fig. 4a, the signal intensity obtained for all conjugates was higher in Skov-3 tumours compared to xenografted A549 tumours, except for **3**, presumably due to high background signal. A significant difference in average radiance between Skov-3 and A549 was obtained for conjugate **2** (*p* < 0.05) (Fig. 4b). FRα immunoreactivity score (0–8) was assessed by IHC in xenografted Skov-3 (4.6 ± 2.5) and A549 (2.7 ± 0.8) tumours (Fig. 4c), confirming an association between higher fluorescence intensities in Skov-3 tumours and higher target expression, which was also previously observed by flow cytometry and *in vitro* FLI (Fig. 2 and S18). However, the standard deviation demonstrates heterogenous expression of FRα in both Skov-3 and A549 xenografts, as already demonstrated for other models [28] (Fig. 2a and 4c).

*Ex vivo* fluorescence analysis of tumour tissues reveals signal in all Skov-3 tumours except **4**, with a lower overall intensity in A549 (Fig. 4d). The biodistribution to the kidneys of conjugates **1**, **2** and **3** suggests higher hydrophilicity, translated into renal clearance (Fig. 4d and e). Consistently low fluorescent background signals are shown for **4** and **5**, with accumulation of **4** mainly in the liver and residual fluorescence signal of **5** in the stomach (Fig. 4d and e).

**Fig. 5.**
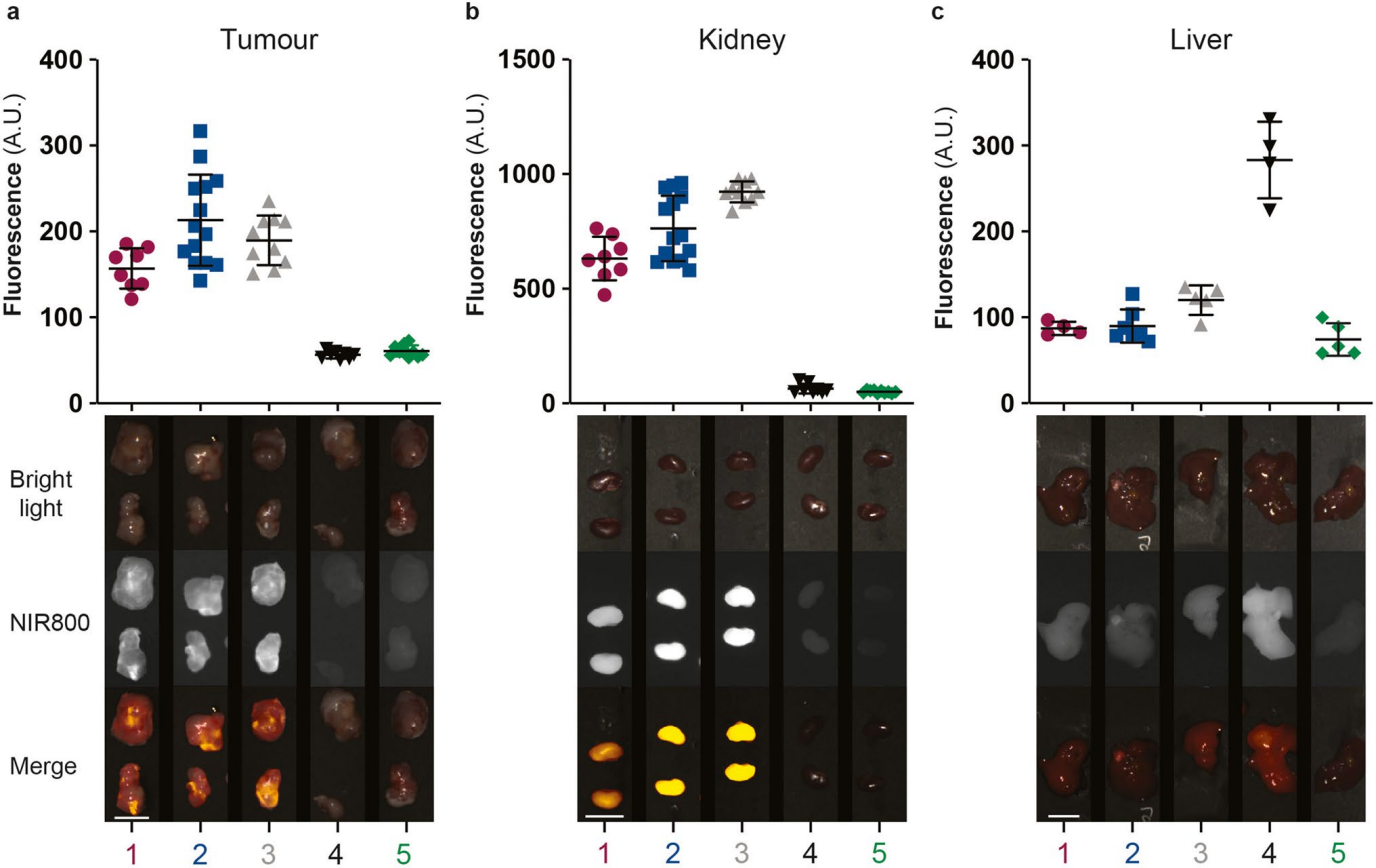
Intraoperative assessment of conjugates in Skov-3 xenografts. *Ex vivo* fluorescence intensities for (**a)** tumour (2000 ms exposure time), (**b)** kidney (1000 ms exposure time), and (**c)** liver (2000 ms exposure time) presented in arbitrary greyscale units (A.U.) (**1** *n* = 8; **2** *n* = 14; **3** *n* = 10; **4** *n* = 8; **5** *n* = 10, at 4h for **1**–**3** and 24h for **4** and **5**). Representative colour, near-infrared (NIR) 800 and pseudo-coloured fluorescent signal merge image of the subcutaneous tumour, kidney and liver. (Scale bars 1 cm).

Finally, the fluorescence of all conjugates was evaluated using a clinically compatible intraoperative NIR-FLI system, to assess their suitability as contrast agents for FIGS. The fluorescence signal from Skov-3 tumours, kidneys and liver confirmed the results obtained with the IVIS Spectrum (Fig. 5). As shown in Fig. 5a, the fluorescence signal in tumours, acquired with an exposure time of 2000 ms, was minimal for conjugates **4** and **5** (56.4 ± 4.1 and 60.9 ± 6.3 AU, respectively), rendering them ineffectual for *in vivo* evaluation of FRα expression. In addition, we observed low fluorescence in the kidneys (100.4 ± 37.8 and 63.9 ± 11.3 AU, respectively, Fig. 5b) for both conjugates, but high fluorescence intensity in the liver of mice injected with **4** (283.3 ± 38.7 AU, Fig. 5c). Tumour-specific fluorescence was observed for conjugate **1 (**156.9 ± 22.0 AU), **2** (213.1 ± 51.0 AU) and **3 (**189.7 ± 27.1 AU) (Fig. 5a). The *ex vivo* comparison of signal intensities in the intraoperative setting of Skov-3 and A549 xenografted tumours revealed increased fluorescence in Skov-3 tumours for all conjugates (Fig. S22), confirming the *ex vivo* results obtained from the IVIS Spectrum. While fluorescence values in the liver were below the ones obtained for the tumours, fluorescence in the kidneys was higher than in the tumours in all cases, with the highest kidney intensities obtained for **3**, 913.6 ± 248.9 AU (Fig. 5b and c).

## Discussion

Substantial differences in the optical and targeting properties of conjugates formed by the same targeting ligand, but with different linkers or fluorophores, have been described [26, 28]. In addition, the choice of fluorophore has a major impact on tumour contrast due to its influence on biodistribution and clearance [31]. This means that the physicochemical properties of the fluorophore also need to be taken into consideration, in addition to the fluorescent optical properties such as large Stokes shifts, high extinction coefficient, quantum yield and serum stability. Based on these observations, we investigated the impact of the fluorescent dye properties of conjugates **1**–**5** on tumour targeting and biodistribution. We aimed to identify the superior fluorophores for further development of fluorescent conjugates for FIGS, and our results suggest that ZW800-1 Forte is a potential candidate for conjugation to small molecule targeting ligands.

The fluorophores in this study differ with respect to net charge, hydrophilicity and spectral properties. ZW800-1 and ZW800-1 Forte, a more stable analogue of the former with a C-C bond instead of the labile ether linkage on the *meso* carbon, are zwitterions that exhibit a balanced net charge of zero. This characteristic is expected to reduce non-specific binding to, e.g., serum proteins, and thus reduce the background signal [3, 32]. Indeed, zwitterionic NIR fluorophore-based targeted tracers have been shown to have favourable *in vivo* imaging characteristics, such as low background signal and rapid renal clearance in several preclinical studies [33], and have been clinically evaluated in patients with colon carcinoma [34]. In our study, conjugate **2** (ZW800-1 Forte) exhibited an intense fluorescence signal in tumours shortly after intravenous injection that was sustained for up to 48 h and it was the only conjugate that demonstrated significant differences between Skov-3 and A549. In contrast, there is a high non-specific fluorescent accumulation of **3** (IRDye® 800CW) at early time points in both cell line models, which obstructs clear distinction of the tumour signal, indicating that background reduction is pivotal for enhancing tumour contrast. IRDye® 800CW contains four sulfonate groups, giving it a high net negative charge. This negative charge results in a highly hydrophilic dye that interferes with the target binding properties of the conjugates, resulting in increased non-specific accumulation of the conjugate [3]. A study comparing IRDye® 800CW and ZW800-1, using cRGD as the target in preclinical melanoma models, shows similar tumour signal intensities, but a significant increase in TBR for the zwitterionic conjugate [26]. ICG-OSu and Cy7 possess one and no sulfonate group, respectively, and are thus more hydrophobic, which affects the clearance and circulation times of the folate conjugate [3]. Rapid renal clearance rates were observed *in vivo* for conjugates **1**, **2** and **3**, leading to a decrease in the fluorescence signal of FRα negative tissues [17, 33, 35]. ICG-OSu-based conjugate **4** showed no accumulation in tumours and high biodistribution in the liver. This is also observed for the highly hydrophobic-free dye ICG, which is known to bind to plasma proteins, eliminating contrast agents larger than 8 nm via the hepatobiliary route [36, 37]. In addition, ICG exhibits limited photostability, which could explain the overall low fluorescence signal intensities obtained with ICG-OSu [38]. In contrast, conjugate **5** began to accumulate in the subcutaneous tumour 4 h after administration, reaching maximum fluorescence signal intensity at 24 h. We hypothesise that this could be the result of an unspecific EPR effect, following binding of **5** to albumin due to the presence of the *meso*-chlorine on the cyclohexenyl moiety [39]. The undesired low fluorescence intensity of **5** further limits its applicability for FIGS development. Lastly, conjugation of the different fluorophores to EDAF resulted in distinct absorption shifts with moderate bathochromic shifts for conjugates **1**–**4** and a hypsochromic shift for conjugate **5** in culture medium, compared to the literature values for the free dyes.

Optimal tumour contrast is critical for FIGS and can be achieved with both low background fluorescence, through reduction of non-specific binding, and high tumour-specific fluorescent signal, through improved targeting. Fluorescent conjugates targeting FRα are employed in late-stage clinical studies and have demonstrated favourable contrast for ovarian cancer FIGS, leading to improved surgical outcome [8, 40, 41]. However, in our study, the overall low TBR values obtained in subcutaneous ovarian cancer models could be due to the intermediate number of folate receptor antigen binding sites in the chosen cell line, Skov-3, or a potentially low binding affinity of the small molecule.

Distinct dyes exhibit excitation and emission spectra that require different *in vivo* imaging settings. While the preclinical IVIS Spectrum allows the user to adjust the excitation and emission filters, most intraoperative imaging systems do not have this function. Therefore, it is important to consider both the properties of the FIGS imaging system and the contrast agent. In our study, the properties of the intraoperative camera (λ_ex_ = 760 ± 3 nm, λ_em_ = > 781 nm) are most favourable for conjugate **2** (λ_ex_ = 760 nm, λ_em_ = 788 nm) compared to the other conjugates, which have higher excitation wavelengths. Thus, the right combination of fluorophore, targeting ligand, linker and imaging system is necessary for increased tumour sensitivity and specificity, to ultimately achieve superior optical contrast.

## Conclusions

Our results confirm that the physicochemical properties of fluorophores have a major impact on the biodistribution of the conjugates and the subsequent tumour contrast. The IRDye® 800CW conjugate **3** showed a high *in vivo* background fluorescence and was not sensitive enough to assess the differences in FRα expression between Skov-3 and A549. Although IRDye® 800CW is the most commonly employed agent for clinical use, we have demonstrated that other fluorescent contrast agents should be taken into consideration. ZW800-1 Forte has demonstrated potential as fluorescent contrast agent for targeted strategies providing specific fluorescence signal.

## Supplementary Information

ESM 1 (DOCX 7364 kb)
