# Supplementary material for: Comparison of Five Near-Infrared Fluorescent Folate Conjugates in an Ovarian Cancer Model
Source: Mol Imaging Biol. 2021 Dec 10;25(1):144–55. doi: 10.1007/s11307-021-01685-y (PMC9971101; doi:10.1007/s11307-021-01685-y)
Supplement: Supplementary file 1 — (DOCX 7364 kb) [file 11307_2021_1685_MOESM1_ESM.docx]

**Electronic Supplementary Material**

Comparison of five near-infrared fluorescent folate conjugates in an ovarian cancer model.

Elvira García de Jalón^1,2*^, Katrin Kleinmanns^1*^, Vibeke Fosse^1^, Ben Davidson^3^, Line Bjørge^1,4^, Bengt Erik Haug^2§^ and Emmet McCormack^1,5,6§^

*^1^Centre for Cancer Biomarkers CCBIO, Department of Clinical Science, The University of Bergen, Jonas Lies vei 65, Bergen 5021, Norway; ^2^Department of Chemistry and Centre for Pharmacy, University of Bergen, Allégaten 41, N-5007, Bergen, Norway; ^3^Department of Pathology, Oslo University Hospital, Norwegian Radium Hospital, and Faculty of Medicine, Institute of Clinical Medicine, University of Oslo, Oslo, Norway; ^4^Department of Obstetrics and Gynecology, Haukeland University Hospital, 5021 Bergen, Norway; ^5^Centre for Pharmacy, Department of Clinical Science, The University of Bergen, Jonas Lies vei 65, Bergen 5021, Norway; ^6^Vivarium, Department of Clinical Science, The University of Bergen, Jonas Lies vei 65, 5021 Bergen, Norway.*

*** The authors contributed equally to this work

| *^§^Corresponding Authors:* | **First Authors:* |
| --- | --- |
| *Professor Emmet Mc Cormack* | *Dr. Elvira García de Jalón, M.Sc.* |
| *Tel: +47 55 97 3097* | *Tel: + 47 94434599* |
| *Email:* [*emmet.mc.cormack@uib.no*](mailto:emmet.mc.cormack@uib.no) | *Email: elvira.vioegra@uib.no* |
| *Professor Bengt Erik Haug* | *Dr. Katrin Kleinmanns* |
| *Tel: +47 55 58 34 68* | *Tel: + 47 96703136* |
| *Email:* [*Bengt-Erik.Haug@uib.no*](mailto:emmet.mc.cormack@uib.no) | *Email: Katrin.Kleinmanns@uib.no* |

**Content overview Supporting information:**

Synthesis of folic acid (FA) dye conjugates: 3

Materials and instruments: 7

Methods: 8

Synthesis of 10: [6] 8

Synthesis of 12 (Pteroic acid): [4] 8

Synthesis of 11: 9

Synthesis of 7: [7] 10

General protocol for conjugates 1 to 5: 10

Synthesis of 1: 11

Synthesis of 2: 12

Synthesis of 3: 13

Synthesis of 4: 14

Synthesis of 16: 14

Synthesis of 17: 15

Synthesis of 5: 16

Analytical data: 17

Figure S1: ^1^H-NMR spectrum of 12 in DMSO 17

Figure S2: ^1^H-NMR spectrum of 11 in DMSO 18

Figure S3: ^1^H-NMR spectrum of EDAF 7 in DMSO 19

Figure S4: HRMS spectrum of EDAF 7 20

Figure S5: Analytical RP-HPLC chromatogram of 1 20

Figure S6: HRMS spectrum of 1 21

Figure S7: Analytical RP-HPLC chromatogram of 2 22

Figure S8: HRMS spectrum of 2 23

Figure S9: HRMS spectrum of 3 24

Figure S10: Analytical RP-HPLC chromatogram of 4 25

Figure S11: HRMS spectrum of 4 26

Figure S12: ^1^H-NMR spectrum of 16 in DMSO 27

Figure S13: HRMS spectrum of 16 28

Figure S14: Analytical RP-HPLC chromatogram of 5 29

Figure S15: HRMS spectrum of 5 30

Figure S16: Physicochemical properties of conjugates 1 – 5. 31

Figure S17: Comparative of the optical properties of five far-red shifted folate-dye conjugates a) in medium and b) in DMSO. 32

Figure S18: Binding affinity and competition assay 33

Figure S19: WST-1 cell metabolic activity assay. 33

Figure S20: Assessment of conjugate biodistribution in subcutaneous Skov-3 xenograft models. 34

Figure S21: HPLC trace and MS data of the corresponding HPLC trace for conjugate 1 35

Figure S22: Intraoperative tumour signal from subcutaneous Skov-3 and A549 xenografts

(FLARE) 35

References: 36

# Synthesis of folic acid (FA) dye conjugates:

Bettio *et al.* have reported that the FRα-binding affinities of α- and γ-isomers of FA-conjugates were in the same range and that a mixture can be used *in vivo* [1]*.* On the other hand, Muller *et. al.* have showed in biodistribution studies that the γ-isomer displays more favourable kinetics [2]. We have therefore explored into different synthetic routes to obtain the pure γ-isomer of the FA-dye conjugates **1** - **5**. As all the dyes used in this study contain an NHS-ester to facilitate conjugation to biomolecules through amide bond formation by reaction with the side chain of lysine side chains, we aimed at using ethylenediamine as a linker between FA and the dyes. Toward this end, *N*-Boc protected **6** was prepared following a literature procedure (see Scheme S1) [3].

Upon direct activation of FA with *N*-hydrosuccinimide (NHS) (1.0 or 0.9 equiv.) in the presence of either of the carbodiimides DIC (1.0 equiv.) or DCC (0.9 equiv.), unreacted FA was the major component that could be isolated (Scheme S1). To increase the conversion, the reaction was performed with 2 equiv. of NHS and 2 equiv. of the carbodiimide, only to give the bis-activated α-γ isomer or unreacted FA as major products with DIC or DCC, respectively. Subsequent reaction of the crude mixture with *N*-Boc protected ethylenediamine (**6**) and DIPEA yielded a mixture of the α-, γ- and α-γ isomers. Purification prior to removal of the Boc-group allowed for removal of the bis-ethylenediamine derivative. Subsequent deprotection and purification using RP-HPLC gave the desired γ-ethylenediamine folate (**7**) in high purity (≥95%).

Scheme S1. Attempted syntheses of EDAF (7) directly from folic acid. 8: α-isomer and 9: α-γ isomer.

We also tested the direct coupling of FA with **6** (4 equiv.) using DCC (0.5, 1.0 or 2.0 equiv.) as the activating agent, however this also gave unreacted FA in the first two cases or bis-functionalised FA as for the NHS approach.

In order to avoid the challenging purification steps required to separate **7**, **8** and **9**, we decided to pursue a selective synthesis of **7** by starting out with pteroic acid (**12**, Scheme S2). First, FA was converted to **12** in a two-step process following a published method [4] (Scheme S2). Next, the appropriately protected glutamic acid part was then pursued by coupling **6** to Fmoc-Glu-O*t*Bu, followed by deprotection of the Fmoc-group to give **11** in modest yield. Coupling of **6** to the protected amino acid was also attempted using DCC and NHS, however this required an extra purification step. Finally, coupling of **11** and **12** using HATU as the coupling reagent (use of HCTU or EDCI/HOBt proved unsuccessful) followed by global deprotection and purification using RP-HPLC gave the desired γ-ethylenediamine folate (**7**) in high purity (≥95%), albeit in low yield. While this work was ongoing, the necessity of a stepwise approach to synthesise the pure γ-regioisomer of folate has also been described by Figliola *et al.* [5].

Scheme S2. Synthesis of γ-ethylenediamine folate (EDAF, 7) from pteroic acid (12).

As shown in Scheme S3, one-pot condensation of the Fischer bases **13** and **15** (synthetic details provided in manuscript under peer review) with **14** (synthesised following a published method) successfully gave substrate **16**. The reaction also gave the undesired symmetric side products, which resulted in an overall low yield (19%) for the target dye after purification. Subsequent reaction of **16** with TSTU and DIPEA, followed by purification using RP-HPLC gave **17** in modest yield (26%).

Scheme S3. Synthesis of the dye and its corresponding NHS ester for conjugate 5.

Finally, conjugation of **7** with the NHS esters of the different Cy7 dyes in dry DMSO at rt for 24 h using either DIPEA or TEA as base (Scheme S4), gave conjugates **1** – **5** after purification by RP-HPLC (Table 1).

Scheme S4. Synthesis of EDAF-Cy7 conjugates (1 - 5).

Synthesis of **3** using DIPEA was unsuccessful and TEA was employed instead. In addition, to purify the conjugates with high presence of sulfonate groups on them, ammonium acetate (10 mM) was added to the mobile phase employed for RP-HPLC to promote the formation of ion pairs and improve the separation. Conjugate **5** was purified using 0.1% TFA (Table 1).

Table 1: Summary of yield and purity after preparative RP-HPLC for each conjugate (1 – 5)

| Conjugate | Amount (mg) | Yield (%) | Purity (%, by HPLC) |
| --- | --- | --- | --- |
| **1** | 0.68 | 57 | 100 |
| **2** | 0.35 | 29 | 100 |
| **3** | 2.55 | 60 | - |
| **4** | 0.87 | 60 | 93 - 100 |
| **5** | 0.42 | 13 | 97 |

For conjugate **1**, the fraction EGJV-359 I F1 exhibits a small shoulder indicating the presence of unreacted dye as confirmed by MS analysis. Hence, conjugate **1** employed for the *in vivo* experiments might have contained minor quantities of free dye.

# Materials and instruments:

Compound **6** [3], **13** [6]**, 14** [7] and **15 [6]** were prepared following published methods. All reactants and reagents employed in the synthetic work were obtained from Sigma-Aldrich and were used as received. Reactions were generally carried out under argon atmosphere using overnight oven-dried (130 °C) equipment that was cooled down under reduced pressure and purged with argon prior to use. Dry THF was obtained from an anhydrous solvent delivery system (SPS-800 system from M. Braun GmbH, Garching, Germany) and stored in a Schlenk flask under argon. Anhydrous DMF (99.8%), anhydrous DMSO (≥ 99.9%) and anhydrous acetonitrile (99.8%) were purchased from Sigma-Aldrich (Cat. No. 227056, Cat. No. 276855 and Cat. No. 271004, respectively) and stored under argon.

Flash chromatography was performed using silica (Silica gel 60, 0.040 – 0.063 mm, Merck) using manually packed glass columns or using Grace^TM^ Reveleris^TM^ SRC C18 cartridges (40 µm, 40g, Grace Discovery Sciences, Maryland, USA) on a Puriflash XS 420 system (Interchim, Montlucon Cedex, France). Preparative high-performance liquid chromatography (HPLC) was performed on a Gilson 321 multisolvent pump with a Dionex Ultimate 3000 variable wavelength detector using an Ascentis C18 (250 x 21.2 mm, 100 Å, 5 μm) column with mixtures of acetonitrile and water (both containing 0.1% TFA or 20 mM of ammonium bicarbonate) as eluent.

Analytical HPLC was performed on a 1290 Infinity II Flexible pump with a 1260 Infinity II DAD WR detector (Agilent, Santa Clara, USA) using a ZORBAX RRHD Eclipse plus 300-SB C18 (50 x 2.1 mm, 300 Å, 1.8 μm) column with mixtures of acetonitrile and water (both containing 0.1% TFA or 10 mM of ammonium acetate) as eluent. High-resolution mass spectra were recorded with an AccuTOF^TM^ mass spectrometer (Agilent, Santa Clara, USA) operated with an orthogonal electrospray ionization (ESI) source, an orthogonal accelerated time of flight (TOF), single stage reflectron mass analyser and a dual micro channel plate (MCP) detector. NMR spectra were recorded using either a Bruker BioSpin AV500 and/or a Bruker BioSpin Ascend spectrometer (Bruker, Billerica, USA) operating at 850 MHz with an inverse-detected triple resonance (TCI) cryoprobe for ^1^H NMR and 2D NMR experiments. ^1^H chemical shifts (δ) are reported in ppm with reference to the solvent residual peak (δ_H_ 2.50 for (CD_3_)_2_SO). All coupling constants are given in Hz.

# Methods:

## 1-(4-(((2-amino-4-oxo-3,4-dihydropteridin-6-yl)methyl)amino)benzoyl)-5-oxopyrrolidine-2-carboxylic acid (10): [8]

Folic acid (447 mg, 1.0 mmol) was dissolved in anhydrous THF (5 mL) and cooled in an ice/water bath. Trifluoroacetic anhydride (1.1 mL, 7.9 mmol) was added dropwise over 30 min after which the cooling bath was removed and stirring continued at rt overnight. The resulting mixture was evaporated to give a brown viscous residue that was redissolved in THF (20 mL) and ice (25 g) was added before the mixture was stirred for 5 h and then poured into stirring diethyl ether (95 mL). The precipitate that formed was isolated by filtration and dried under high vacuum to give the title compound as a light peach orange solid (405 mg, 96% crude yield). The crude material was taken to the next step without further purification.

## 4-(((2-amino-4-oxo-3,4-dihydropteridin-6-yl)methyl)amino)benzoic acid (Pteroic acid) (12): [4]

Intermediate **10** (3.475 g, 8.21 mmol) was dissolved in concentrated HCl (120 mL) and the resulting mixture was heated at 60 °C in an oil bath overnight. After cooling to rt, stirring was continued for 3 h. The precipitate that formed was isolated by filtration, dried under high vacuum and it was used in the next step without any further purification.

Beige-greenish solid; 1.456 g (57% crude yield). ^1^H-NMR (500 MHz, (CD_3_)_2_SO): δ = 8.76 (s, 1H), 8.58 (s, 2H), 7.65 (m, 2H), 6.65 (m, 2H), 4.59 (s, 2H).

## *Tert*-butyl N^5^-(2-((tert-butoxycarbonyl)amino)ethyl)-L-glutaminate (11):

*Step 1:* Fmoc-Glu-O*t*Bu (0.226 g, 0.5 mmol) and HCTU (0.218 g, 0.5 mmol) were dissolved in dry DMF (3 mL), DIPEA (0.17 mL) was added, and the resulting mixture was stirred at rt for 10 min followed by addition of **7** (96 mg, 0.6 mmol) dissolved in dry DMF (1 mL). The resulting mixture was stirred at rt for 6 h and then diluted with ethyl acetate (15 mL). This mixture was washed with 10% citric acid (15 mL), water (15 mL), 10% NaHCO_3_ (15 mL), water (15 mL) and saturated NaCl (15 mL). The organic phase was dried over MgSO_4_, filtered and evaporated under reduced pressure to give the title compound (131 mg, 46% crude yield), which was used in the next step without any further purification.

*Step 2:* To a solution of the material from the previous step (606 mg, 1.1 mmol) in dichloromethane (5 mL), a solution of diethylamine (2 mL) in dichloromethane (10 mL) was added dropwise. The resulting mixture was stirred at rt for 2 h, after which the mixture was evaporated under reduced pressure to give an oily residue (585 mg). The crude product was purified by flash column chromatography (ethyl acetate/methanol, 4:1 v/v) as eluent) to give the title compound.

Colourless oil; 311 mg (51%). ^1^H NMR (500 MHz, (CD_3_)_2_SO): δ = 7.82 (t, *J* = 5.7, 1H), 6.77 (t, *J* = 5.7, 1H), 3.17 (dd, *J* = 7.8, 5.7, 1H), 3.04 (q, *J* = 6.3, 2H), 2.95 (q, *J* = 6.3, 2H), 2.13 (t, *J* = 7.8, 2H), 1.78 (m, 1H), 1.58 (m, 1H), 1.41 (s, 9H), 1.37 (s, 9H).

## *N*^2^-(4-(((2-amino-4-oxo-3,4-dihydropteridin-6-yl)methyl)amino)benzoyl)-*N*^5^-(2-aminoethyl)-*L*-glutamine (7): [9]

*Step 1:* Glutamic acid derivative **11** (86 mg, 0.25 mmol) was dissolved in dry DMF (2 mL) and added to a mixture of **7** (81 mg, 0.25 mmol), HATU (95 mg, 0.25 mmol) and DIPEA (0.15 mL, 0.83 mmol) and the resulting mixture was stirred at rt under an argon stream overnight. Water (5 mL) was added and the precipitate that formed was isolated by centrifugation and dried under high vacuum to give a sticky orange solid (103 mg, 64% crude yield), which was used in the next step without any further purification.

*Step 2:* Boc-protected ethylenediamine folate (354 mg, 0.55 mmol) was dissolved in TFA (6 mL) and the mixture was stirred at rt for 90 min. The brownish solution was treated with diethyl ether (15 mL) and the precipitate that formed was isolated by centrifugation and dried under high vacuum to give the title compound. The brown solid residue obtained was purified using semi-preparative RP-HPLC (1 - 40% acetonitrile in water, both solvents containing 0.1% formic acid) to give the title compound.

Yellow solid: 19 mg (6% yield). ^1^H-NMR (850 MHz, (CD_3_)_2_SO): δ = 8.66 (s, 1H), 8.23 (d, *J* = 8.0, 1H), 7.97 (t, *J* = 5.7, 1H), 7.69 (bs, 3H), 7.66 (d, *J* = 9.0, 2H), 6.65 (d, *J* = 9.0, 2H), 4.50 (s, 2H), 4.37 (m, 1H), 3.32 (m, 1H), 3.18 (m, 1H), 2.83 (m, 2H), 2.20 (m, 2H), 2.12 (m, 1H), 1.88 (m, 1H). The spectrum contains small additional peaks. ESI-LRMS: Calcd m/z for C_21_H_26_N_9_O_5_^+^ [M + H]^+^: 484.2; found: 484.3.

## General protocol for conjugation to NHS-esters (1 – 5):

A solution of **7** (1 equiv.) in dry DMSO (500 µL) was added to a mixture of the NHS ester (1 equiv./ 2 equiv.) of the corresponding dye and DIPEA (3 equiv.) or TEA (3 equiv.) in dry DMSO and the resulting mixture was stirred at rt in the dark for 24 h. The reaction mixture was purified by semi-preparative HPLC.

## 2-((*E*)-2-((*E*)-2-(4-(3-((2-((*S*)-4-(4-(((2-amino-4-oxo-3,4-dihydropteridin-6-yl)methyl)amino)benzamido)-4-carboxybutanamido)ethyl)amino)-3-oxopropyl)phenoxy)-3-(2-((*E*)-3,3-dimethyl-5-sulfonato-1-(3-(trimethylammonio)propyl)indolin-2-ylidene)ethylidene)cyclohex-1-en-1-yl)vinyl)-3,3-dimethyl-1-(3-(trimethylammonio)propyl)-3*H*-indol-1-ium-5-sulfonate (1):

The title compound was prepared from **7** (1.63 mg, 3.4 x 10^-3^ mmol) and ZW800-1 NHS ester (6.0 mg, 5.1 x 10^-3^ mmol) using DIPEA as base following the general protocol.

Light green solid: 1.12 mg (28% yield). ESI-MS: Calcd m/z for C_72_H_92_N_13_O_13_S_2_^2+^ [M + H]^2+^: 704.8145; found: 705.3172.

## 2-((*E*)-2-((*E*)-4'-(3-((2-((*S*)-4-(4-(((2-amino-4-oxo-3,4-dihydropteridin-6-yl)methyl)amino)benzamido)-4-carboxybutanamido)ethyl)amino)-3-oxopropyl)-6-(2-((*E*)-3,3-dimethyl-5-sulfonato-1-(3-(trimethylammonio)propyl)indolin-2-ylidene)ethylidene)-3,4,5,6-tetrahydro-[1,1'-biphenyl]-2-yl)vinyl)-3,3-dimethyl-1-(3-(trimethylammonio)propyl)-3*H*-indol-1-ium-5-sulfonate (2):

The title compound was prepared from **7** (1.7 mg, 3.4 x 10^-3^ mmol), ZW800-1 Forte NHS ester (4.0 mg, 3.4 x 10^-3^ mmol) and DIPEA following the general protocol. Product obtained by precipitation with diethyl ether and decantation.

Light green solid; 1.51 mg (32% yield). ESI-MS: Calcd m/z for C_72_H_91_N_13_O_12_S_2_^2+^ [M]^2+^: 1393.6341, 696.8170; found: 1393.6352. 697.4765.

## 1-(6-((2-((*S*)-4-(4-(((2-amino-4-oxo-3,4-dihydropteridin-6-yl)methyl)amino)benzamido)-4-carboxybutanamido)ethyl)amino)-6-oxohexyl)-2-((*E*)-2-((*E*)-3-(2-((*E*)-3,3-dimethyl-5-sulfonato-1-(4-sulfonatobutyl)indolin-2-ylidene)ethylidene)-2-(4-sulfonatophenoxy)cyclohex-1-en-1-yl)vinyl)-3,3-dimethyl-3*H*-indol-1-ium-5-sulfonate (3):

The title compound was prepared from **7** (1.41 mg, 2.9 x 10^-3^ mmol), IRDye 800CW NHS ester (5.0 mg, 5.8 x 10^-3^ mmol) and TEA following the general protocol.

Green solid; 2.55 mg (60% yield). ESI-MS: Calcd m/z for C_67_H_79_N_11_O_19_S_4_^2+^ [M + H]^2+^: 735.2252; found: 735.2569. Calcd m/z for C_67_H_78_N_11_O_19_S_4_^2+^ [M]^+^: 1468.4353; found: 1468.4360.

## 4-(2-((1*E*,3*E*,5*E*,7*Z*)-7-(3-(6-((2-((*S*)-4-(4-(((2-amino-4-oxo-3,4-dihydropteridin-6-yl)methyl)amino)benzamido)-4-carboxybutanamido)ethyl)amino)-6-oxohexyl)-1,1-dimethyl-1,3-dihydro-2*H*-benzo[*e*]indol-2-ylidene)hepta-1,3,5-trien-1-yl)-1,1-dimethyl-1*H*-benzo[*e*]indol-3-ium-3-yl)butane-1-sulfonate (4):

The title compound was prepared from **7** (1.95 mg, 4.0 x 10^-3^ mmol), ICG-Osu (5.0 mg, 6.0 x 10^-3^ mmol) and DIPEA following the general protocol.

Green solid; 2.03 mg (42% yield). ESI-MS: Calcd m/z for C_66_H_74_N_11_O_9_S^+^ [M]^+^: 1196.5386; found: 1196.5460.

## 2-((*E*)-2-((*E*)-3-(2-((*E*)-1-(5-carboxypentyl)-3,3-dimethylindolin-2-ylidene)ethylidene)-2-chlorocyclohex-1-en-1-yl)vinyl)-1,3,3-trimethyl-3*H*-indol-1-ium (16):

Fischer bases **13** (317 mg, 1.0 mmol) and **15** (413 mg, 1.0 mmol), bis-imine **14** (475 mg, 1.3 mmol) and sodium acetate (133 mg, 1.3 mmol) were dissolved in acetic acid (10 mL) and acetic anhydride (2.5 mL). The reaction mixture was stirred at 110 °C in the dark for 6 h under an argon atmosphere. The resulting mixture was concentrated under reduced pressure and the residue was partitioned between water (20 mL) and dichloromethane (20 mL). The aqueous phase was extracted with dichloromethane (2 x 20 mL) and the combined organic phases were dried over MgSO_4_, filtered and evaporated under reduced pressure to give a sticky black residue (976 mg). 59 mg of this material were purified by semi-preparative HPLC (70 - 90% acetonitrile in water, both solvents containing 0.1% TFA) to give the title compound.

Dark green solid; 11.2 mg (19% yield). ^1^H-NMR (850 MHz, (CD_3_)_2_SO): δ = 8.27 (d, *J* = 14.2, 1H), 8.24 (d, *J* = 14.2, 1H), 7.64 (d, *J* = 7.4, 1H), 7.62 (d, *J* = 7.4, 1H), 7.46 (m, 2H), 7.43 (m, 2H), 7.30 (td, *J* = 7.2, 1.3, 1H), 7.27 (ddd, *J* = 7.4, 5.6, 2.7, 1H), 6.34 (d, *J* = 14.1, 1H), 6.29 (d, *J* = 14.1, 1H), 4.19 (t, *J* = 7.4, 2H), 3.70 (s, 3H), 2.74 – 2.69 (m, 4H), 2.21 (t, *J* = 7.3, 2H), 1.86 (p, *J* = 6.4, 2H), 1.73 (m, 2H), 1.67 (s, 6H), 1.67 (s, 6H), 1.56 (p, *J* = 7.3, 2H), 1.40 (m, 2H); LRMS (ESI-MS) *m/z* calcd. for C_37_H_44_ClN_2_O_2_^+^ [M]^+^: 583.3086; found: 583.3090.

## 2-((*E*)-2-((*E*)-2-chloro-3-(2-((*E*)-1-(6-((2,5-dioxopyrrolidin-1-yl)oxy)-6-oxohexyl)-3,3-dimethylindolin-2-ylidene)ethylidene)cyclohex-1-en-1-yl)vinyl)-1,3,3-trimethyl-3*H*-indol-1-ium (17):

**16** (12.7 mg, 0.02 mmol), TSTU (17. 5 mg, 0.03 mmol) and DIPEA (0.07 mL, 0.04 mmol) were dissolved in 2 mL of anhydrous acetonitrile and the reaction mixture was stirred at rt for 6 h protected from the light. The excess of solvent was removed by rotary evaporation to give a dark green solid residue, which was purified using semi-preparative RP-HPLC (70 - 90% acetonitrile in water, both solvents containing 0.1% TFA) to give the title compound.

Iridiscent blue solid; 3.9 mg (26% yield).

## 2-((*E*)-2-((*E*)-3-(2-((*E*)-1-(6-((2-((*S*)-4-(4-(((2-amino-4-oxo-3,4-dihydropteridin-6-yl)methyl)amino)benzamido)-4-carboxybutanamido)ethyl)amino)-6-oxohexyl)-3,3-dimethylindolin-2-ylidene)ethylidene)-2-chlorocyclohex-1-en-1-yl)vinyl)-1,3,3-trimethyl-3*H*-indol-1-ium (5):

*Step 1:* Cy7 dye **16** (12.7 mg, 0.02 mmol), TSTU (17. 5 mg, 0.03 mmol) and DIPEA (0.07 mL, 0.04 mmol) were dissolved in 2 mL of anhydrous acetonitrile and the reaction mixture was stirred at rt for 6 h protected from light. The excess of solvent was removed by rotary evaporation to give a dark green solid residue, which was purified using semi-preparative RP-HPLC (70 - 90% acetonitrile in water, both solvents containing 0.1% TFA) to give the NHS-ester **17** as an iridiscent blue solid (3.9 mg, 26% yield).

Step 2: Folic acid derivative **7** (1.85 mg, 3.82 x 10^-3^ mmol) was conjugated with NHS-ester **17** (3.90 mg, 5.72 x 10^-3^ mmol.) using DIPEA following the general protocol.

Dark green solid; 0.42 mg (22% yield).

ESI-MS: Calcd m/z for C_58_H_67_ClN_11_O_6_^+^ [M]^+^: 1048.4959; found: 1048.4969.

# Analytical data:

## Figure S1: ^1^H-NMR spectrum of 12 in DMSO

**12**

## Figure S2: ^1^H-NMR spectrum of 11 in DMSO

**11**

## Figure S3: ^1^H-NMR spectrum of EDAF 7 in DMSO

**7**

## Figure S4: LRMS spectrum of EDAF 7


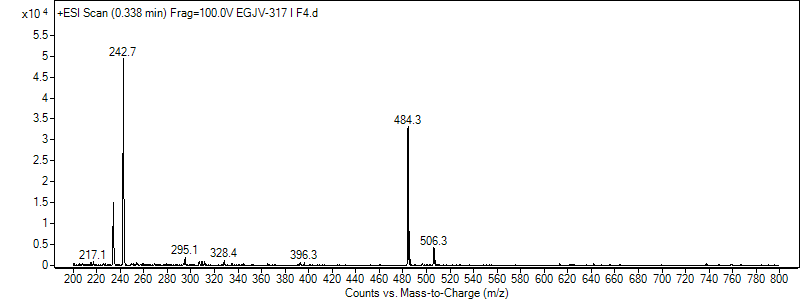


## Figure S5: Analytical RP-HPLC chromatogram of 1

**1**

## Figure S6: HRMS spectrum of 1

**1**

Calcd m/z for C_72_H_91_N_13_O_13_S_2_^2+^ [M+H]^2+^: 704.8145

## Figure S7: Analytical RP-HPLC chromatogram of 2

**2**

## Figure S8: HRMS spectrum of 2

**2**

Calcd m/z for C_72_H_91_N_13_O_12_S_2_^2+^ [M]^2+^: 1393.6341

## Figure S9: HRMS spectrum of 3

**3**

Calcd m/z for C_67_H_79_N_11_O_19_S_4_^2+^ [M+H]^2+^: 734.7213

## Figure S10: Analytical RP-HPLC chromatogram of 4

**4
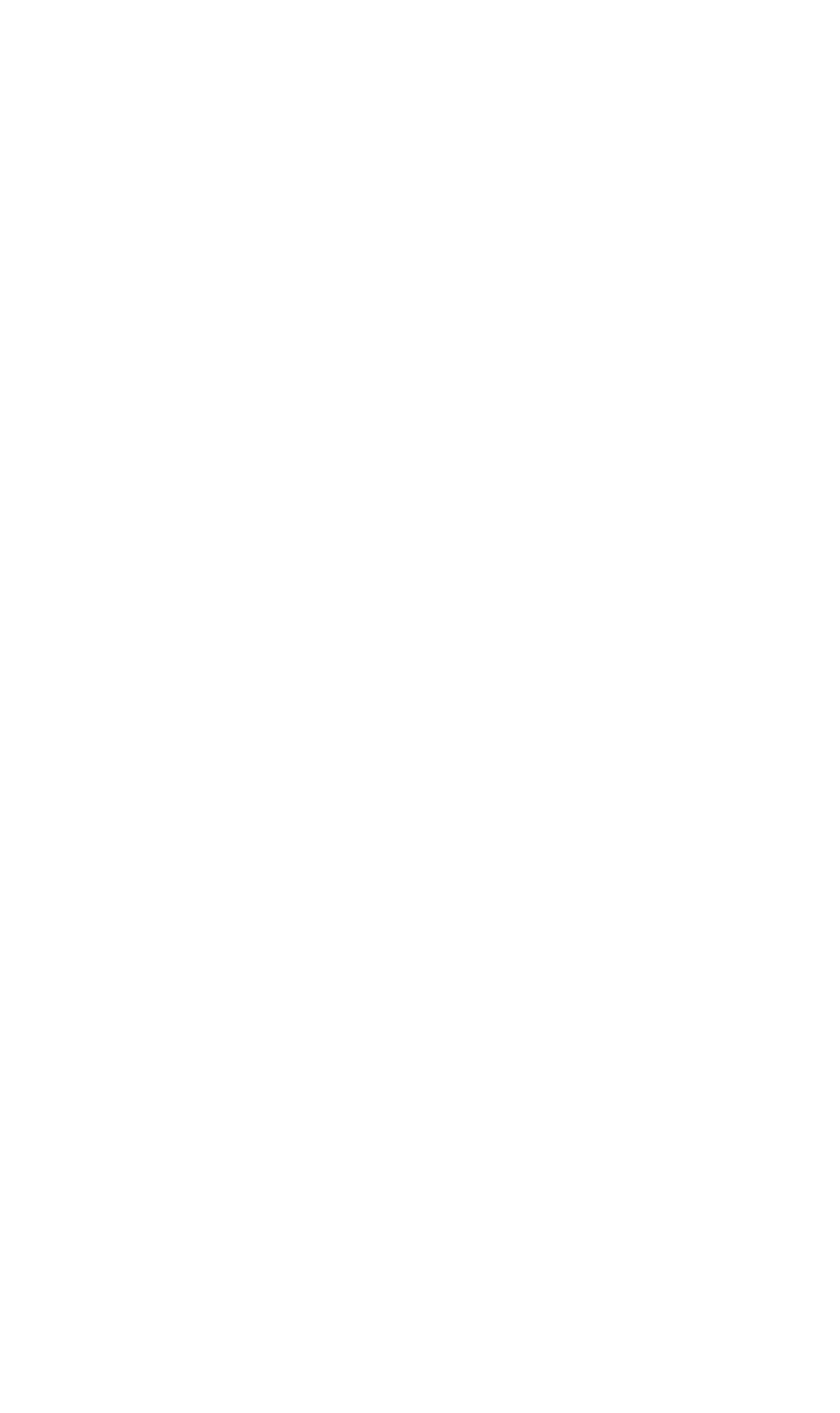
**

## Figure S11: HRMS spectrum of 4

Calcd m/z for C_66_H_74_N_11_O_9_S^+^ [M]^+^: 1196.5386

**4
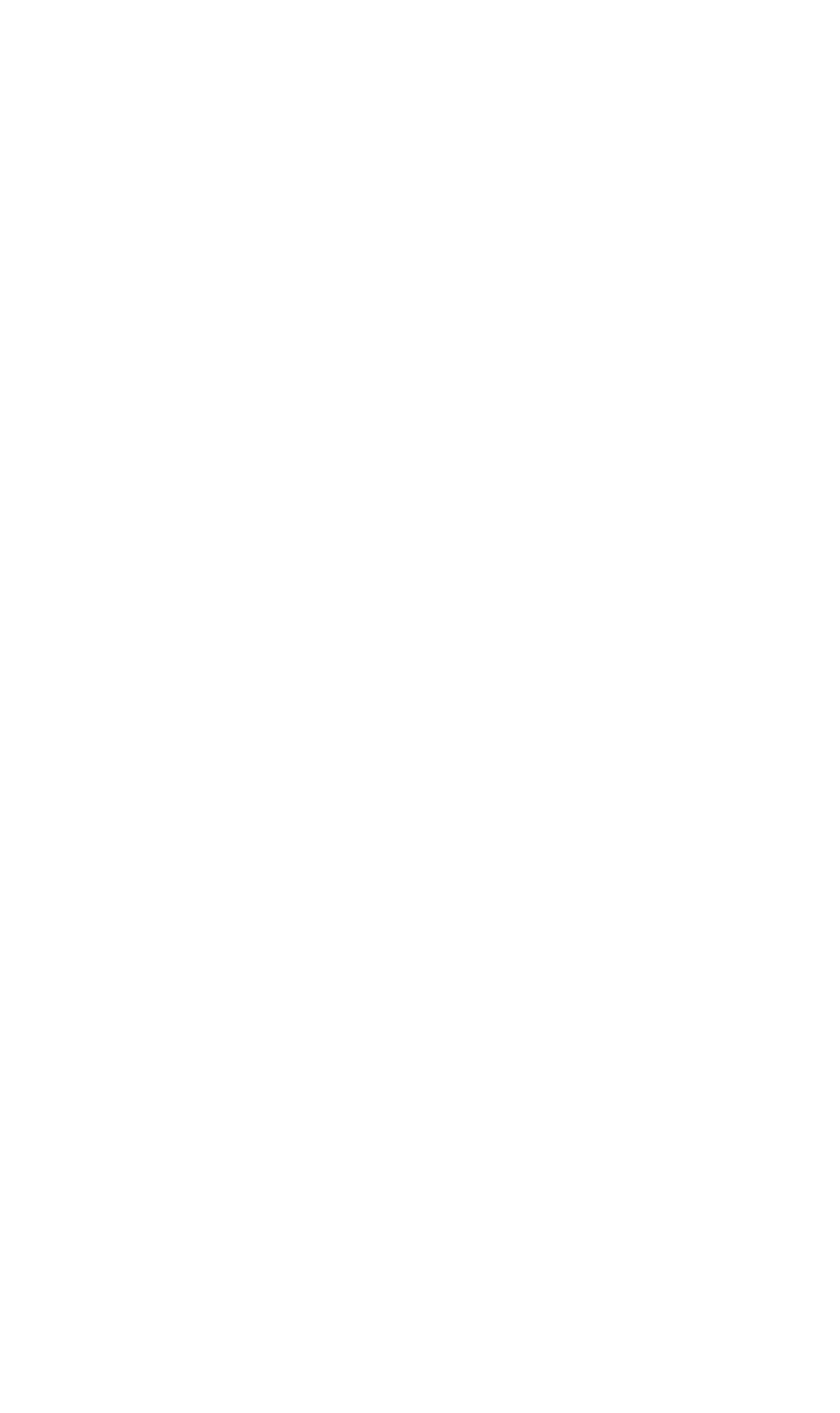
**

## Figure S12: ^1^H-NMR spectrum of 16 in DMSO

##

**16**

## Figure S13: HRMS spectrum of 16

Calcd m/z for C_37_H_44_ClN_2_O_2_^+^ [M]^+^: 583.3086

**16**

## Figure S14: Analytical RP-HPLC chromatogram of 5

**5
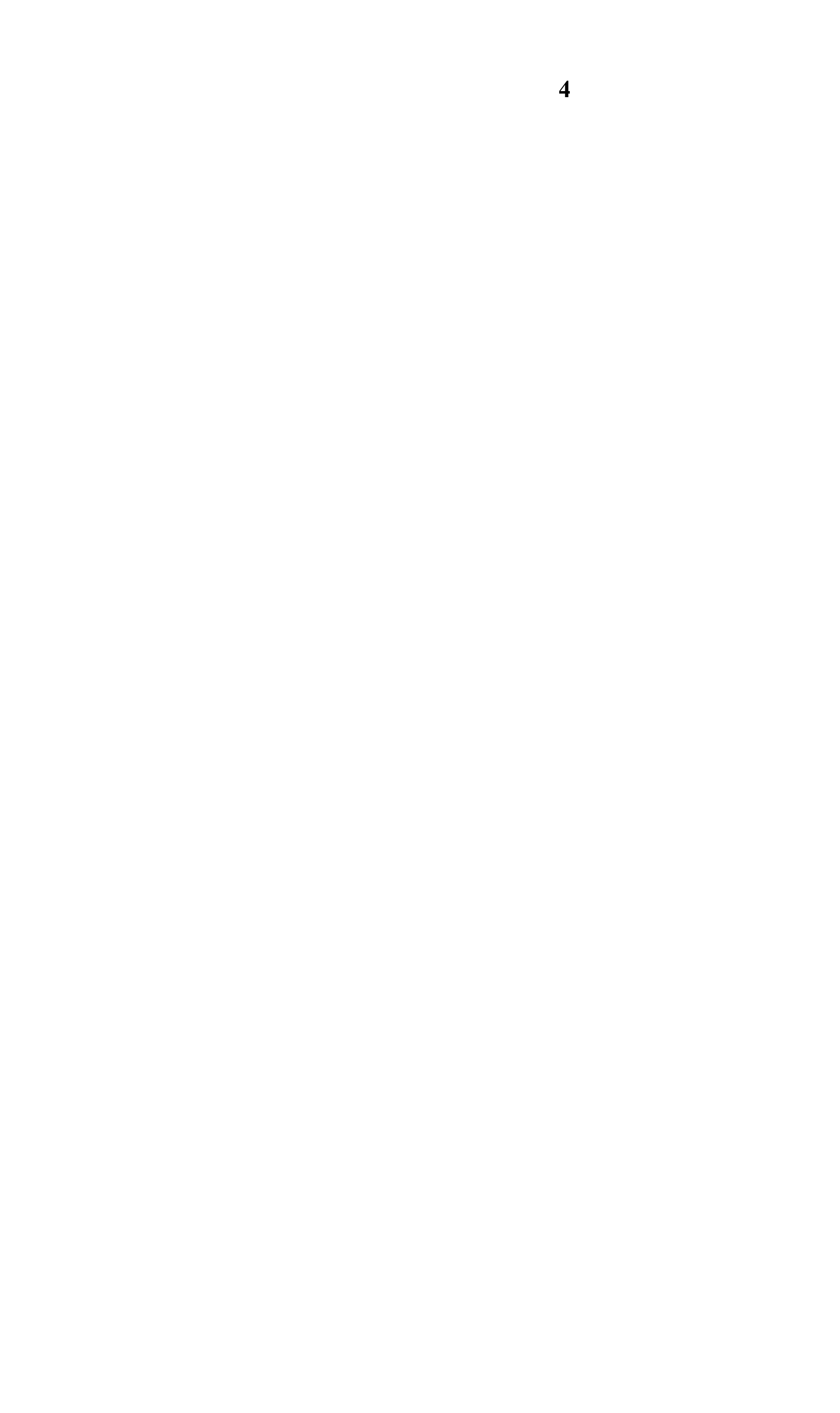

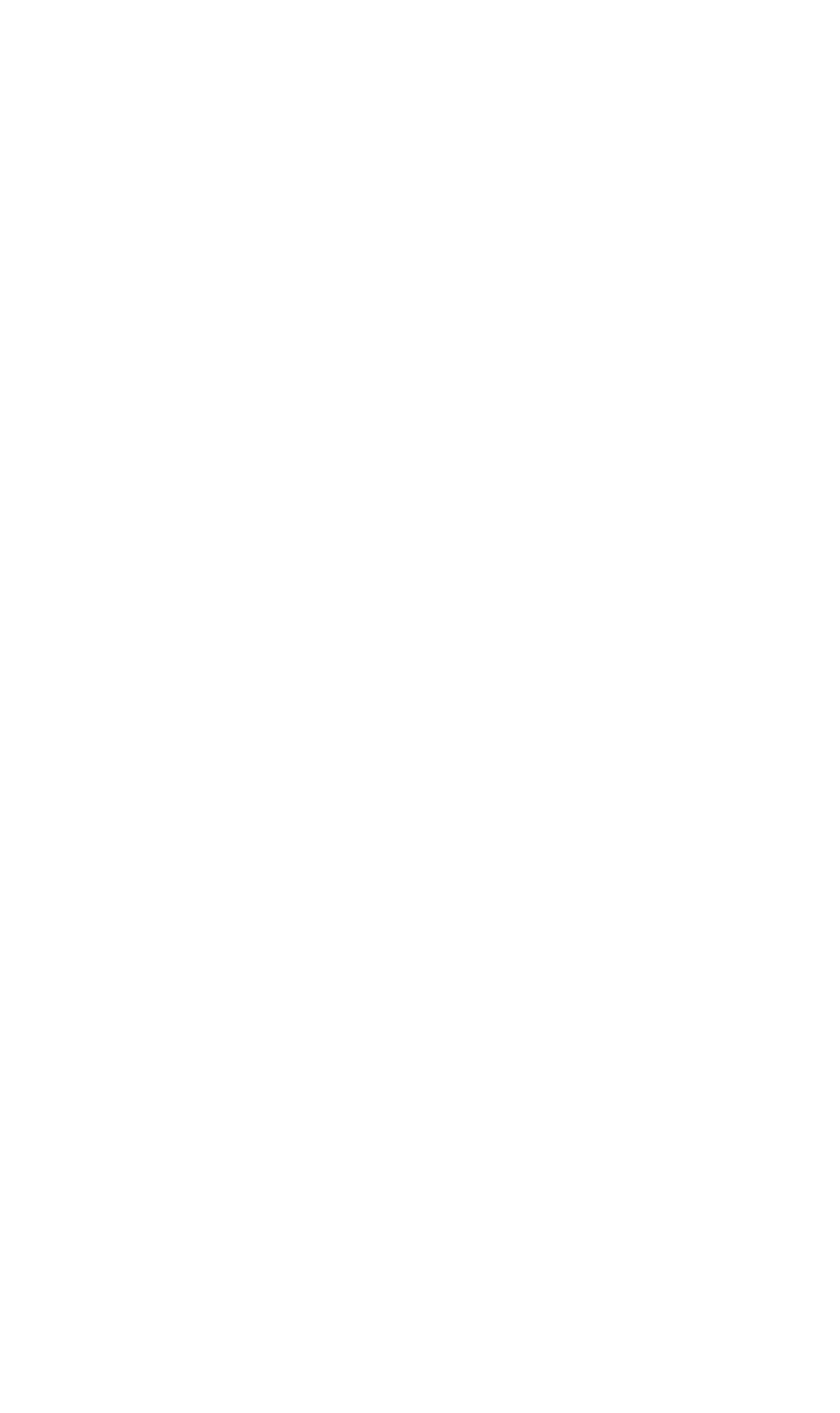
**

## Figure S15: HRMS spectrum of 5

Calcd m/z for C_58_H_67_ClN_11_O_6_^+^ [M]^+^: 1048.4959

**5
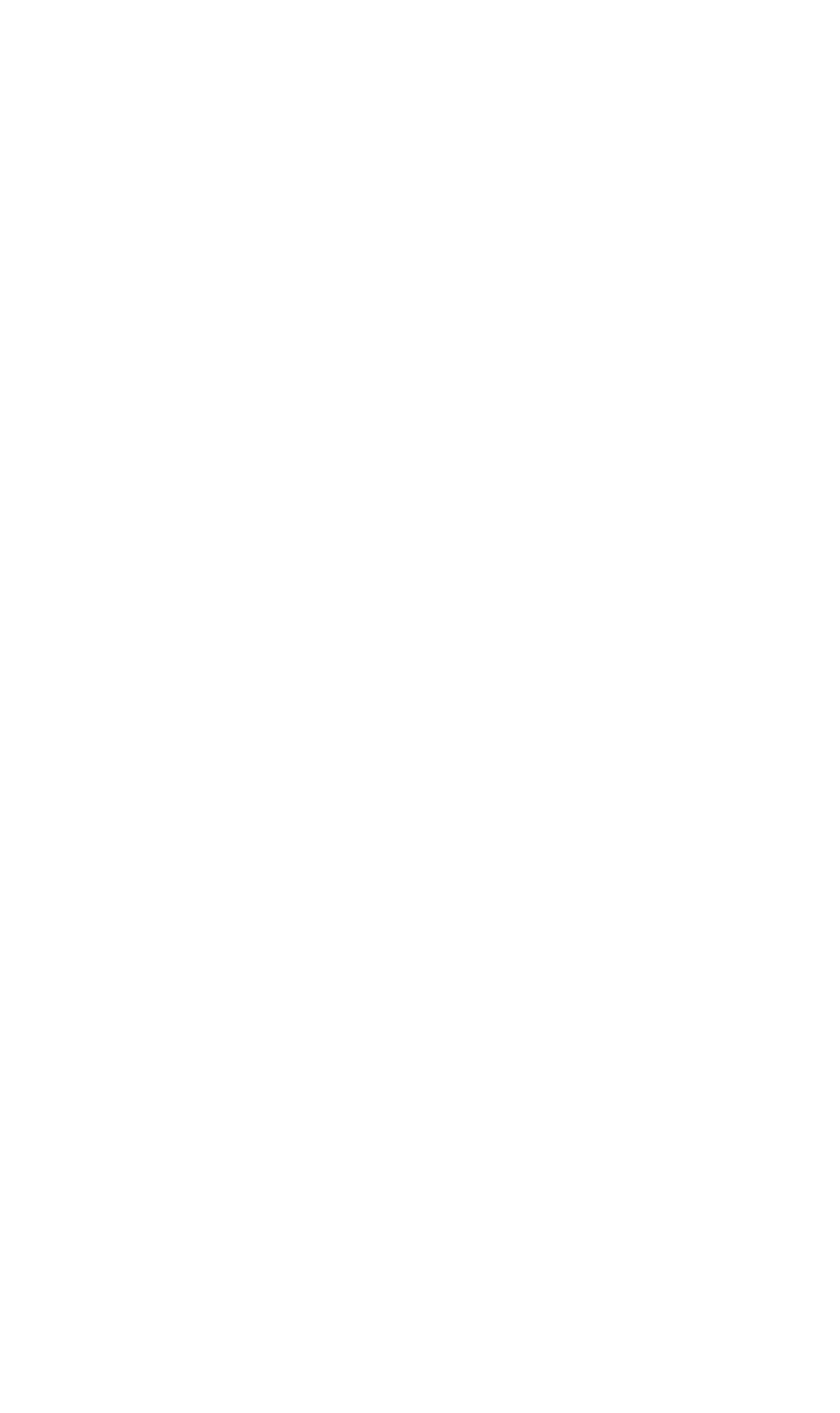
**

## Figure S16: Physicochemical properties of conjugates 1 – 5. Comparison of surface charge distribution (with red and blue representing negative and positive charge, respectively), net charges and LogD at pH = 7.4 for the folate-dye conjugates. This *in silico* data was obtained using MarvinSketch 21.13.0 (Chemaxon).


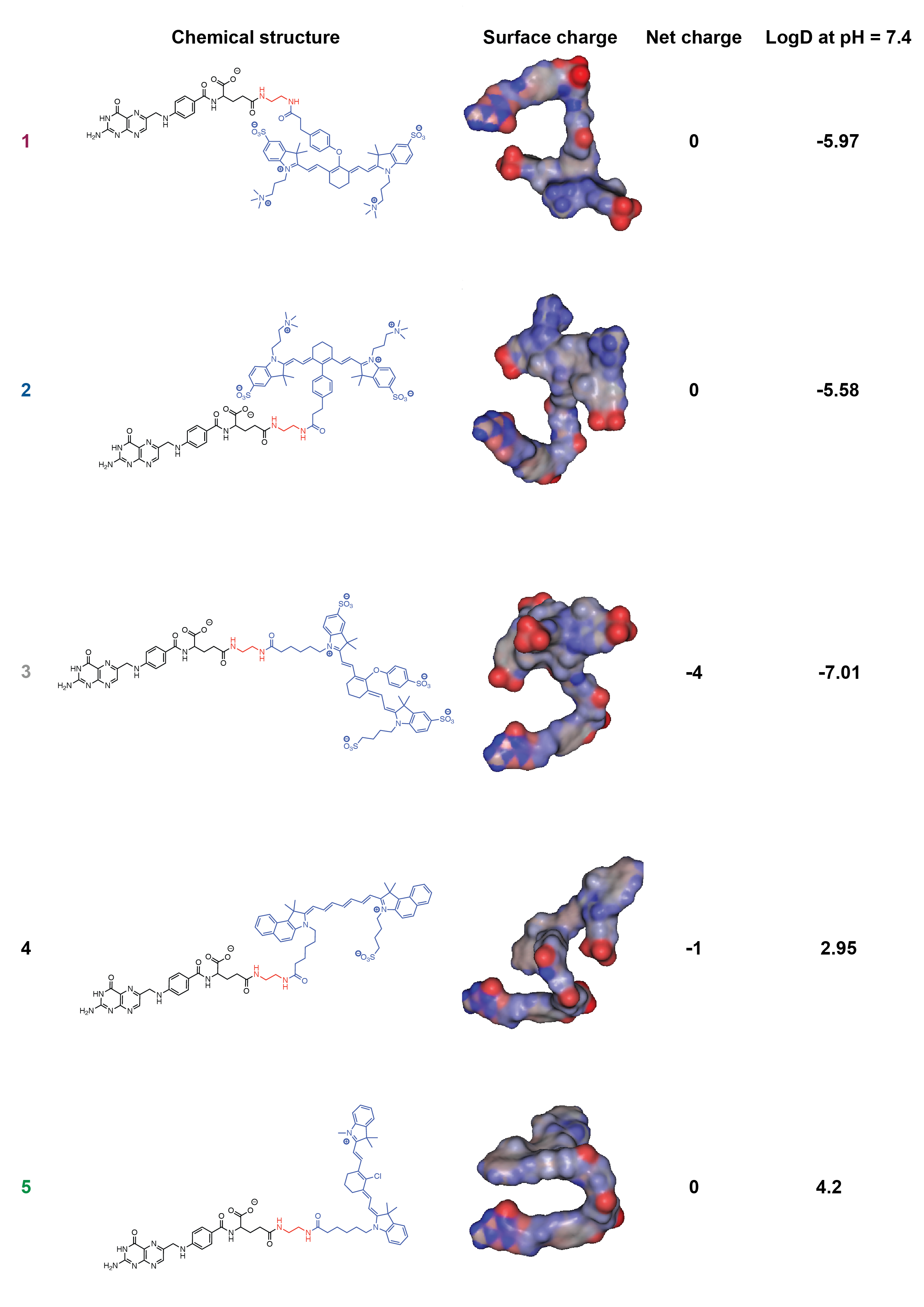


## Figure S17: Comparative of the optical properties of five far-red shifted folate-dye conjugates a) in medium and b) in DMSO.

##
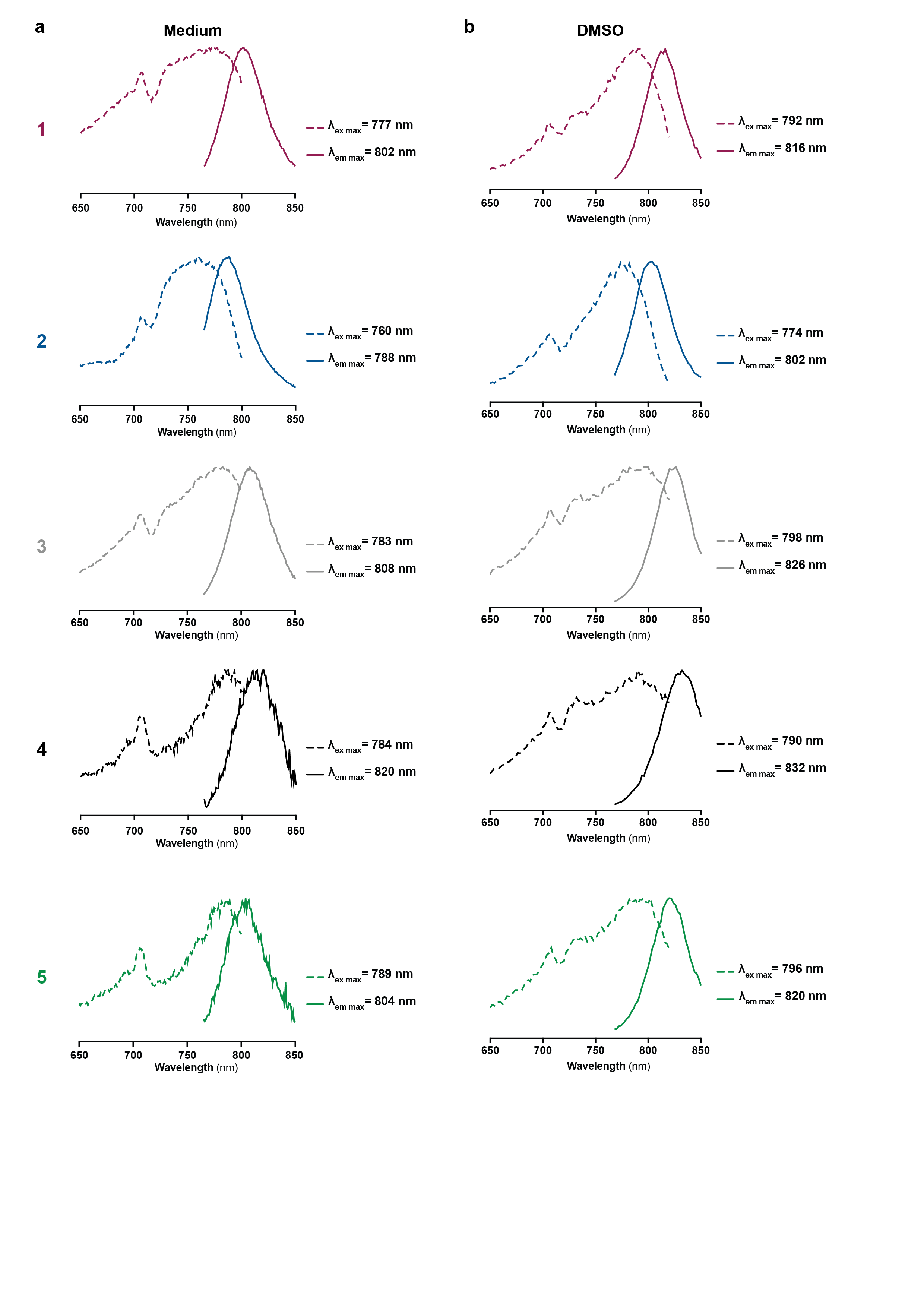


## Figure S18: Binding affinity assay using a serial dilution of 10 μM, 1 μM and 0.1 μM of conjugate 2, in the presence or absence of a 100-fold excess of folic acid, on the study cell line (Skov-3) plus control cell lines A549 (negative control) and HeLa (positive control).


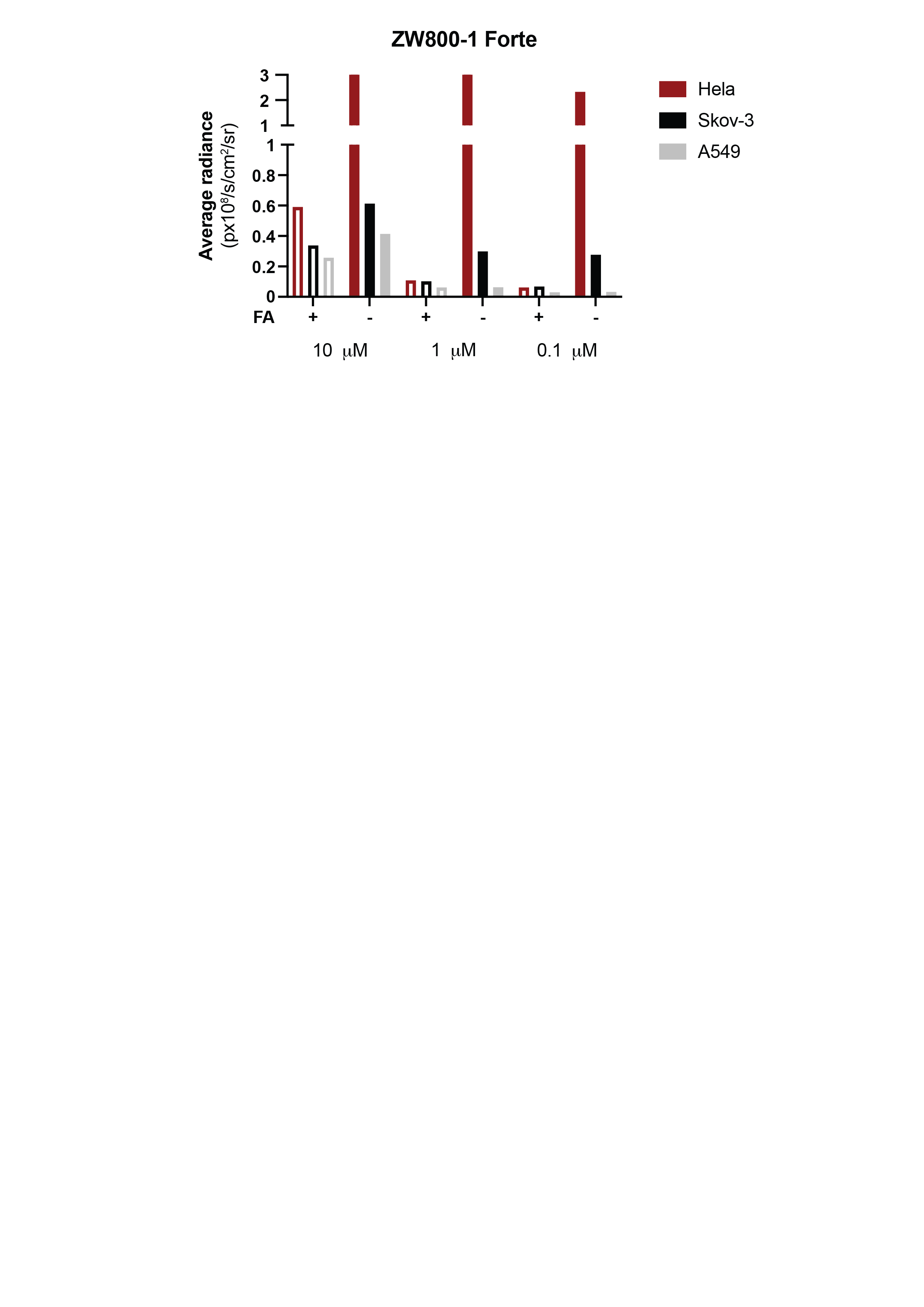


## Figure S19: WST-1 cell metabolic activity assay of Skov-3 cells after incubation with 0.1 - 100 μM of each conjugate (1 – 5) for 4 hours. The spectroscopic signal of formazan from each well incubated with 0.1 - 100 μM conjugate was normalised to unstained control samples. Statistical analysis (one-way ANOVA with unpaired Mann-Whitney U test) with *p* < 0·05 (*) was regarded as statistically significant.


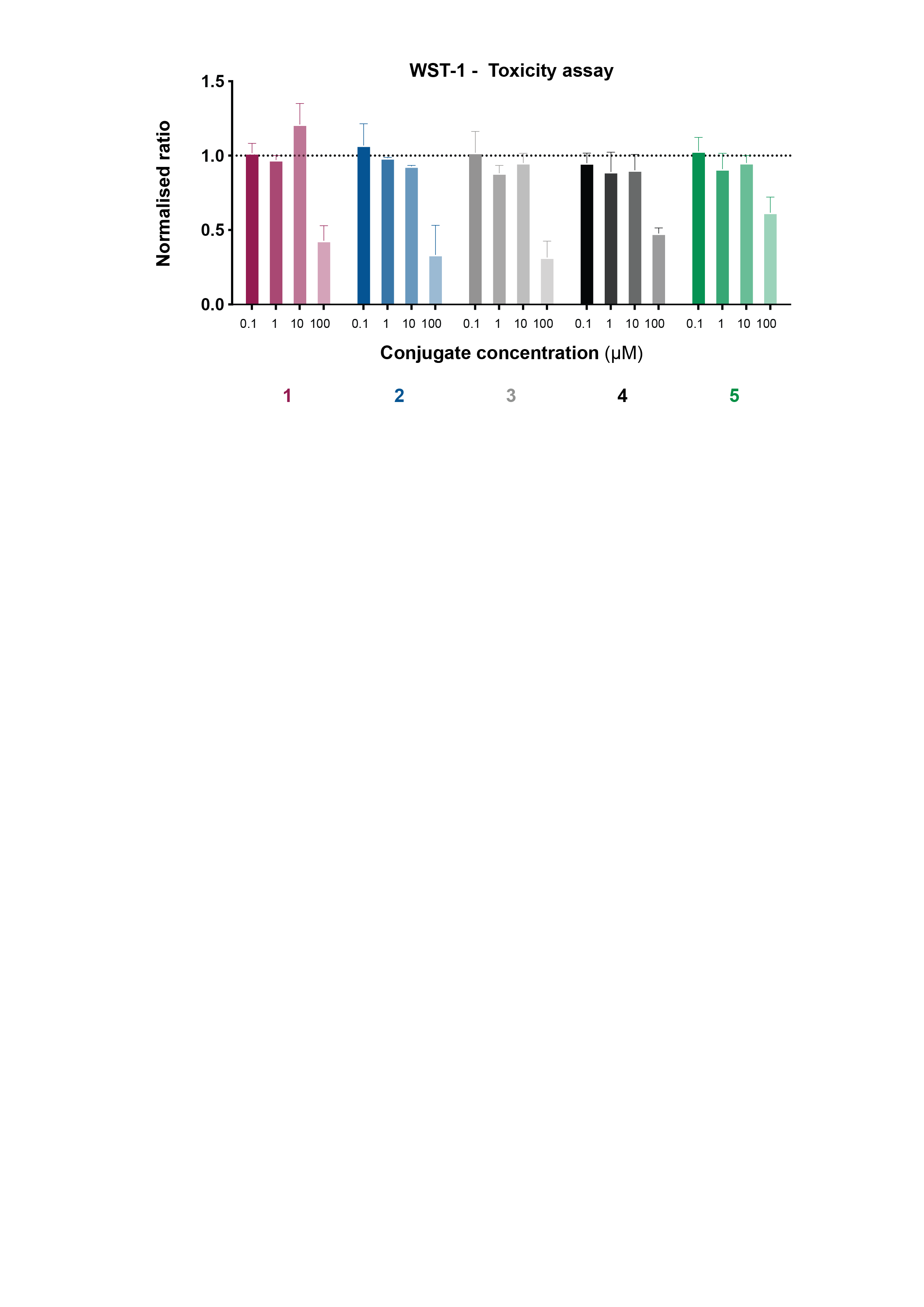


## Figure S20: Assessment of conjugate (1 - 5) biodistribution in subcutaneous Skov-3 xenograft models. Ventral view of longitudinal *in vivo* whole body optical FLI at eight different time points (0.5, 1, 2, 4, 6, 8, 24, and 48 hours) of one representative mouse (n = 3). The fluorescence signal in the bladder or in the liver is highlighted with a dashed white circle for each conjugate 1 - 5 (based on ZW800-1, ZW800-1 Forte, IRDye® 800CW, ICG-OSu and one in-house synthesised dye, respectively).


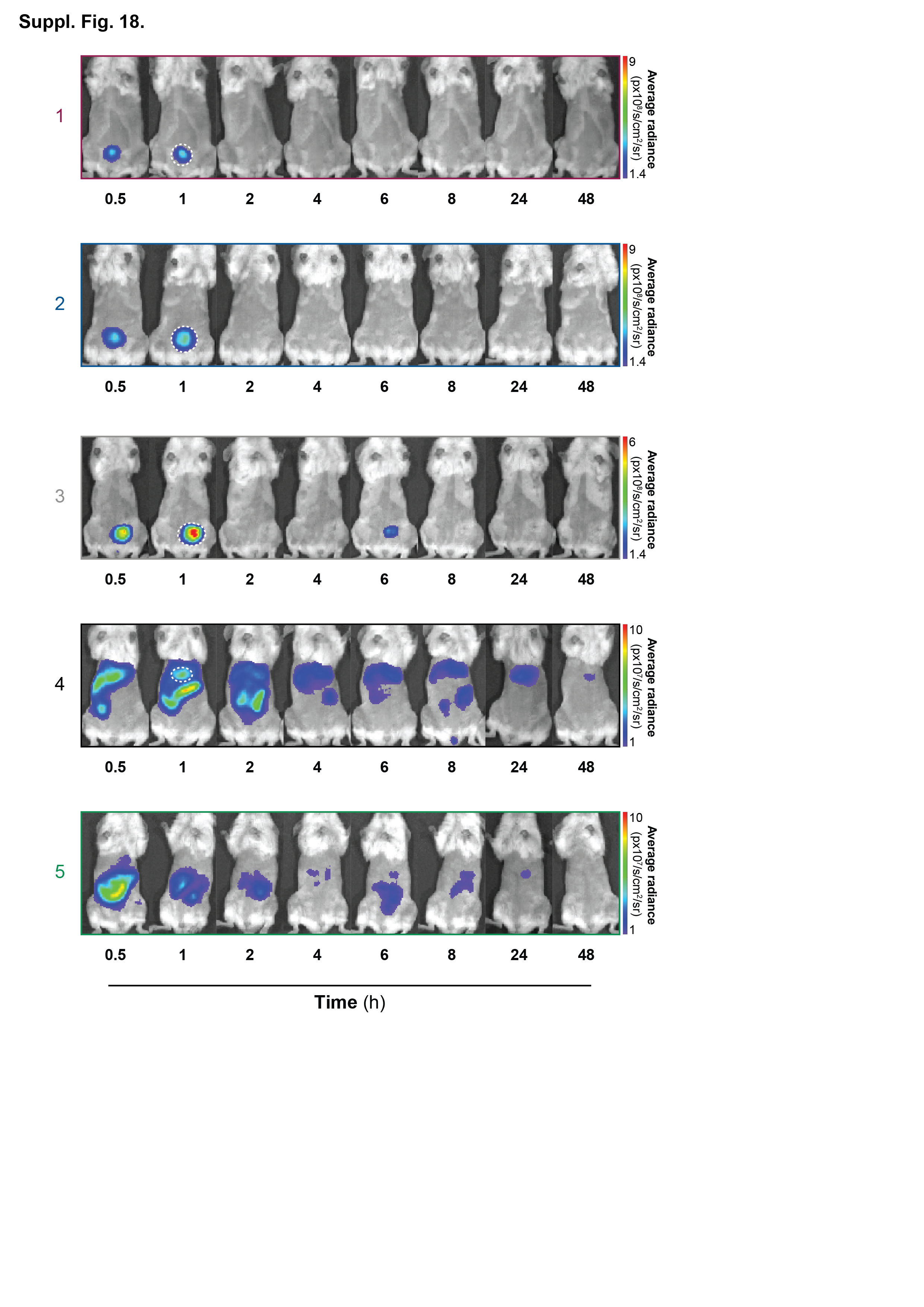


## Figure S21: HPLC trace and MS data of the corresponding HPLC trace for conjugate 1. A shoulder can be observed that corresponds to unreacted dye as confirmed by the MS (*m/z* 315.3).


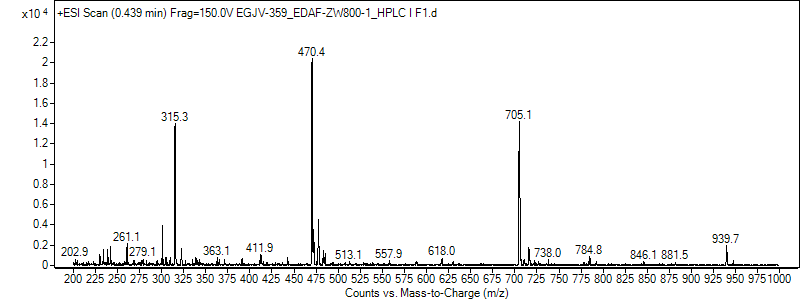


## Figure S22: Intraoperative tumour signal from subcutaneous Skov-3 and A549 xenografts (FLARE)


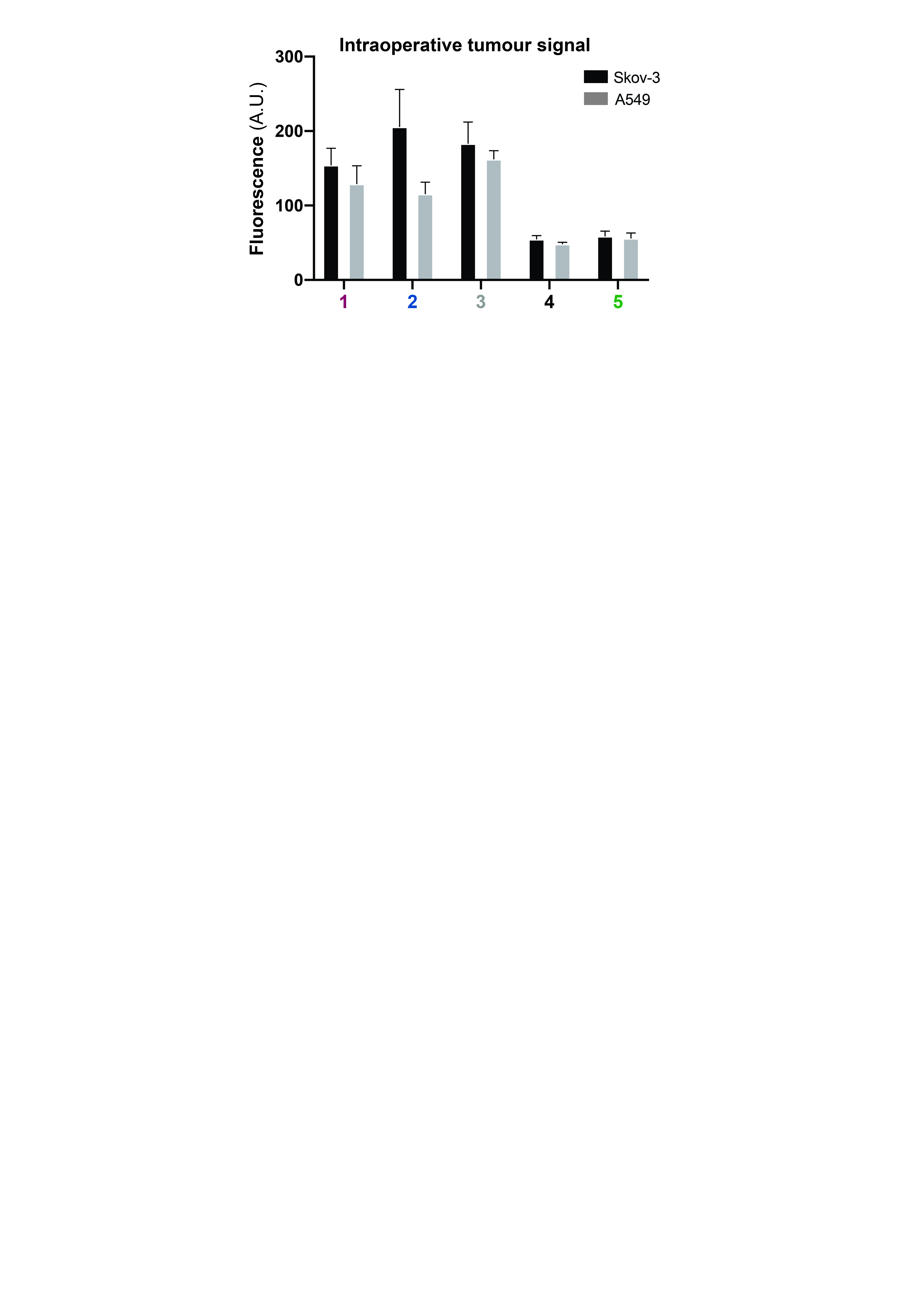


# References:

[1] Bettio A, Honer M, Muller C, Bruhlmeier M, Muller U, Schibli R, et al. Synthesis and preclinical evaluation of a folic acid derivative labeled with 18F for PET imaging of folate receptor-positive tumors. J Nucl Med. 2006;47(7):1153-60.

[2] Muller C. Folate-based radiotracers for PET imaging--update and perspectives. Molecules. 2013;18(5):5005-31.

[3] Holland JP, Fisher V, Hickin JA, Peach JM. Pyrene-Functionalised Copper Complexes as Potential Dual-Modality Imaging Agents. Eur J Inorg Chem. 2010;2010(1):48-58.

[4] Thomas JD, Cui H, North PJ, Hofer T, Rader C, Burke TR. Application of Strain-Promoted Azide–Alkyne Cycloaddition and Tetrazine Ligation to Targeted Fc-Drug Conjugates. Bioconjug Chem. 2012;23(10):2007-13.

[5] Figliola C, Marchal E, Groves BR, Thompson A. A step-wise synthetic approach is necessary to access gamma-conjugates of folate: folate-conjugated prodigiosenes. Rsc Adv. 2019;9(25):14078-92.

[6] García de Jalón E, Ruiz de Garibay G, Haug BE, McCormack E. CytoCy5S™, a compound of many structures. In vitro and in vivo evaluation of four near-infrared fluorescent substrates of nitroreductase (NTR). Under peer review. 2021.

[7] Hyun H, Bordo MW, Nasr K, Feith D, Lee JH, Kim SH, et al. cGMP-Compatible preparative scale synthesis of near-infrared fluorophores. Contrast Media Mol Imaging. 2012;7(6):516-24.

[8] Luo J, Smith MD, Lantrip DA, Wang S, Fuchs PL. Efficient Syntheses of Pyrofolic Acid and Pteroyl Azide, Reagents for the Production of Carboxyl-Differentiated Derivatives of Folic Acid. J Am Chem Soc. 1997;119(42):10004-13.

[9] Port M. (2013). Method for purifying prepared gallium-68 contrast media via column chromatography. (Patent No. FR2980193A1).
